# Effect of Enteral Immunonutrition in Patients Undergoing Surgery for Gastrointestinal Cancer: An Updated Systematic Review and Meta-Analysis

**DOI:** 10.3389/fnut.2022.941975

**Published:** 2022-06-29

**Authors:** Jingyi Shen, Senjie Dai, Zongze Li, Wei Dai, Jiaze Hong, Jin Huang, Jingjie Chen

**Affiliations:** ^1^The Second Clinical Medical College, Zhejiang Chinese Medical University, Hangzhou, China; ^2^Department of General Surgery, HwaMei Hospital, University of Chinese Academy of Sciences, Ningbo, China

**Keywords:** enteral immunonutrition (EIN), gastrointestinal cancer, surgery, complications, meta-analysis

## Abstract

**Background:**

The efficacy of enteral immunonutrition (EIN) in patients undergoing gastrointestinal cancer surgery remains debatable. This meta-analysis aimed to investigate the effectiveness of EIN administration in patients undergoing surgery for gastrointestinal cancer.

**Methods:**

From January 2000 to January 2022, PubMed, EMBASE, Cochrane Library, and Web of Science were thoroughly searched for randomized controlled trials (RCTs) with EIN versus standard diet or no supplement in patients undergoing surgery for gastrointestinal cancer. Overall complications and infectious complications were the primary outcomes. The secondary results were non-infectious complications, mortality, length of hospital stay, and enteral nutrition-related complications.

**Results:**

Thirty-five studies reporting 3,692 patients undergoing surgery for gastrointestinal cancer (including gastric cancer, colorectal cancer, esophageal cancer, periampullary cancer, or pancreatic cancer) were included. Compared with the control group, EIN group had a significantly decreased incidence of overall complications (RR = 0.79, *p* < 0.001). Infectious complications in patients who received EIN were considerably lower than in the control group (RR = 0.66, *p* < 0.001). Compared to the control group, the incidence of surgical site infection, abdominal abscess, anastomotic leakage, bacteremia, duration of systemic inflammatory response syndrome (SIRS), and duration of antibiotic therapy was significantly lower in the specific infectious complications treated with EIN. Still, there was no significant difference between the two groups with other infectious complications. Moreover, a substantial shortening in the length of hospital stay was shown in EIN group compared with the control group. Still, no significant effect of EIN was demonstrated in non-infectious complicatios and mortality. The enteral nutrition-related complications had no significant difference between two groups.

**Conclusions:**

EIN is safe and effective in reducing overall complications, infectious complications, and hospital stay in patients undergoing gastrointestinal cancer surgery (including gastric cancer, colorectal cancer, esophageal cancer, periampullary cancer, or pancreatic cancer).

## Introduction

Gastrointestinal cancers are among the most frequent tumors and a leading cause of cancer death worldwide (1). Compared to other cancer types, gastrointestinal cancer patients have higher malnutrition rates, with the risk of malnutrition reaching up to 80% (2), with a higher risk of upper gastrointestinal cancer (3). Surgery is an essential treatment for gastrointestinal tumors (4–9). Patients undergoing gastrointestinal cancer surgery are at a high risk of poor postoperative outcomes (10, 11). Preoperative malnutrition is an independent risk factor for postoperative complications following gastrointestinal surgery (12–18).

Therefore, nutritional support is essential for patients with gastrointestinal cancer, particularly those undergoing surgery. Enteral immunonutrition (EIN) with specific nutrients such as arginine, glutamine, omega-3 fatty acids, and nucleotides is typically supplemented in formulations (19). EIN can improve nutrition status and enhance immune function (20–24). Some published clinical studies suggested that perioperative EIN administration, enriched with at least two of the immunonutrition nutrients, is beneficial for reducing complications after major abdominal surgery, particularly in malnourished patients (23–27). However, not all studies could draw a similar conclusion; some suggested that EIN does not significantly reduce postoperative complications, mortality, and length of hospital stay (28–30).

There is currently no comprehensive systematic review of the efficacy of perioperative EIN administration in patients undergoing gastrointestinal cancer surgery in literature. Thus, a meta-analysis was conducted to assess the effect of EIN administration vs. control on postoperative outcomes in patients undergoing surgery for gastrointestinal cancer (including gastric cancer, colorectal cancer, esophageal cancer, periampullary cancer, or pancreatic cancer). To fully demonstrate the role of EIN, the study defined EIN as containing at least two or more nutrients, including arginine, glutamine, omega-3 fatty acids, and nucleotides.

## Methods

### Search Strategy

PRISMA 2020 statement: an updated guideline for reporting systematic reviews was used to conduct this systematic review and meta-analysis (31). This meta-analysis investigated was comprehensively conducted in PubMed, EMBASE, Cochrane Library, and Web of Science to search for studies published between January 2000 and January 2022, assessing the impact of EIN on postoperative outcomes, such as complications, in patients undergoing surgery for gastrointestinal cancer. The medical subject heading terms listed below were used and adjusted to meet the requirements of various databases: (immunonutrition OR immune-enhancing nutrition OR immune-enhanced nutrition OR immune-modulating nutrition OR immune nutrition OR immunological nutrition OR glutamine OR omega 3 fatty acid OR ω-3 fatty acid OR n 3 oil OR n 3 fatty acid OR n 3 polyunsaturated fatty acid OR alpha-linolenic acid OR docosahexaenoic acid OR eicosapentaenoic acid OR arginine OR nucleotides) AND (gastrointestinal neoplasm OR gastrointestinal tract cancer OR gastrointestinal cancer OR esophageal neoplasm OR esophagus neoplasm OR esophagus cancer OR esophageal cancer OR intestinal neoplasm OR intestines neoplasm OR intestines cancers OR intestinal cancer OR cecal neoplasm OR cecal cancer OR colorectal neoplasm OR colorectal tumor OR colorectal cancer OR colorectal carcinoma OR duodenal neoplasm OR duodenal cancer OR duodenum cancer OR ileal neoplasm OR ileal cancer OR jejunal neoplasm OR jejunal cancer OR jejunum cancer OR pancreatic neoplasm OR pancreas cancer OR pancreatic cancer OR stomach neoplasm OR gastric neoplasm OR gastric cancer OR stomach cancer). To avoid missing information that might be needed, limitations were not set for the type of specific complications. Relevant bibliographies of identified articles were hand-searched.

### Selection and Exclusion Criteria

The “PICOS” principles were used to develop inclusion and exclusion criteria. There were no restrictions on age, gender, comorbidities, surgical method, or cancer diagnostic criteria. The studies were included if they met the following criteria: (a) participants: patients with gastrointestinal cancer and underwent surgery; (b) intervention: EIN; (c) control: standard diet (an isocaloric and isonitrogenous enteral nutrition supplement) or no supplement (a normal diet without supplements); (d) outcomes: at least one investigated postoperative outcomes, such as complications, mortality, and length of hospital stay; (e) study design: randomized controlled trials (RCTs).

Studies that met any of the following exclusion criteria were excluded: (a) study intervention contained only one component of EIN; (b) articles were not published in English; (c) the data was unavailable. If there are multiple publications from the same trial, the updated or informative article would be used. Two investigators screened titles and abstracts for potentially eligible articles and then retrieved the full text for further selection based on the selection and exclusion criteria.

### Data Extraction

Two investigators extracted data from eligible RCTs independently using a predefined standardized form. Author, year, country, total size, tumor types, time of administration, duration of intervention, EIN composition, infectious complications, non-infectious complications, mortality, length of hospital stay, enteral nutrition-related adverse effects, and the like were among the information gathered. The corresponding authors of studies, or national registry databases used as a data source in the original studies, were consulted for additional information if required. Consensus and discussion were used to resolve any discrepancies.

### Quality Assessment

For assessing the quality of RCTs, the Cochrane Collaboration's tool (32) was used. Random sequence generation (selection bias), allocation concealment (selection bias), blinding of participants and personnel (performance bias), blinding of outcome assessment (detection bias), incomplete outcome data (attrition bias), selective reporting (reporting bias), and other biases were among the domains of bias examined. Bias risk was classified into low risk, unclear risk, and high risk.

### Statistical Analysis

Revman version 5.3 (the Cochrane Collaboration) was used for statistical analysis. A random-effects model was used to assess the postoperative outcomes of gastrointestinal cancer patients undergoing surgery who received EIN or a control group, considering the differences in patient baselines, tumor types, immunonutrition components, and intervention duration. The risk ratio (RR) and 95% confidence interval (95% CI) were applied to analyze dichotomous data. Concurrently, the mean difference (MD) and 95% CI were utilized for the result analysis of continuous data. A two-sided test was used to determine statistical significance, and *p* ≤ 0.05 indicated a statistically significant difference. The chi-squared test and I^2^ test were used to quantify study heterogeneity, classified as low, moderate, high, or severe, corresponding to I^2^ <25%, 25–50%, 50–75%, and >75% (33), respectively. Sensitivity analysis was used to investigate the impact of each study on the overall meta-analysis. The funnel plot identified potential publication bias and the specific causes of publication bias.

## Results

### Eligible Studies

**Figure 1:**
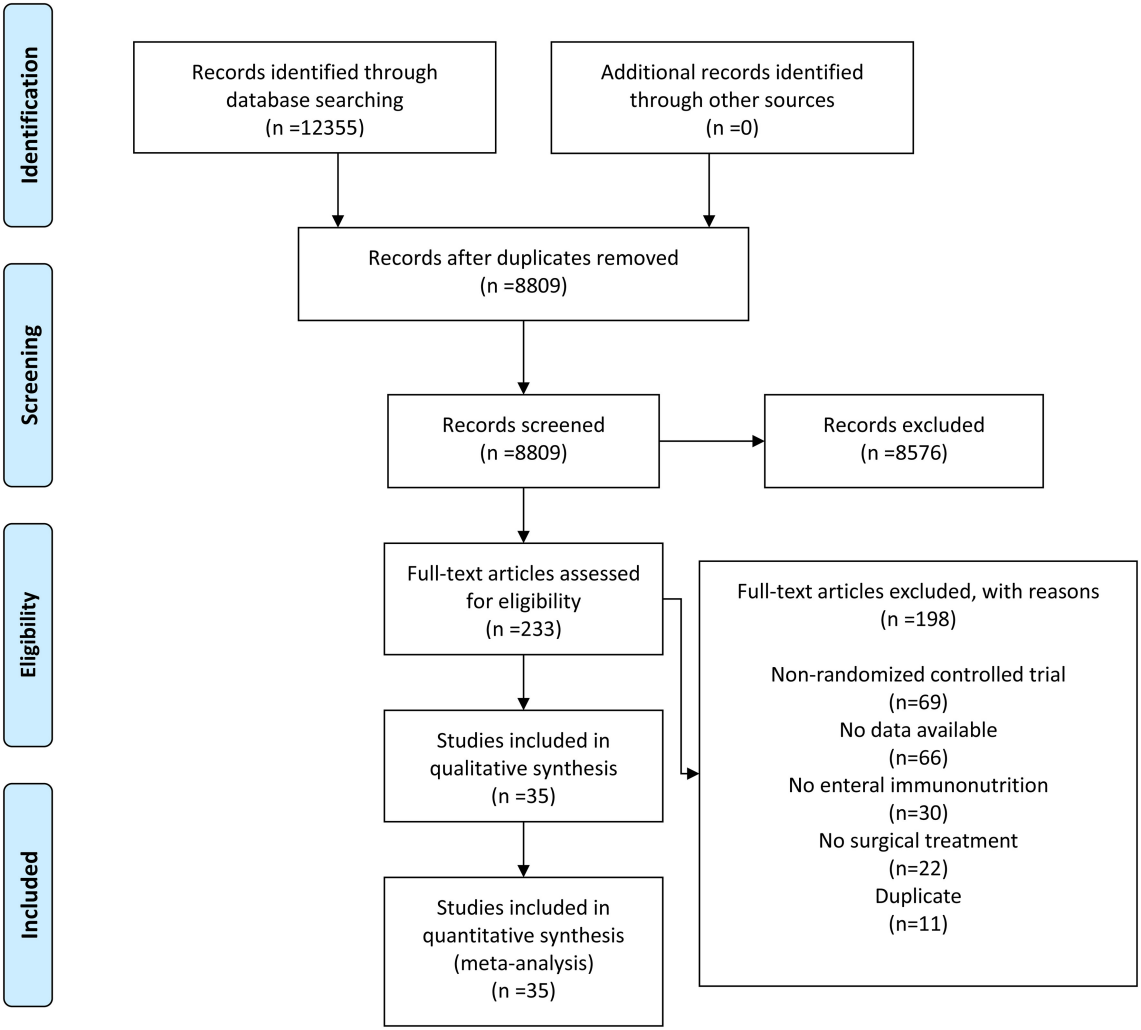
Flow diagram of selection.

The flowchart for the search strategy is displayed in Figure 1. After excluding duplicates and irrelevant records, we identified 233 articles on EIN and gastrointestinal cancers from 12,355 records published between January 2000 and January 2022. By examining the full texts, 198 articles were excluded for non-RCT, no data available, no EIN, no surgical treatment, and duplicate, leaving 35 eligible articles for the final quantitative analysis (23, 24, 26–28, 30, 34–55).

### Study Characteristics and Quality Assessment

Table 1 and Supplementary Table 1 summarize the detailed characteristics of included studies. Nine studies were carried out from Japan (25, 28, 38, 44, 48, 53, 56, 58, 59), six from Poland (26, 30, 39–41, 52), four from Italy (24, 34, 37, 49), four from China (23, 43, 46, 54), three from Spain (45, 50, 51), two from England (22, 36), two from Switzerland (35, 42), two from Turkey (27, 55), one from Denmark (57), one from Korea (47), and one from Australia (29). A total of 3,692 patients undergoing surgery for gastrointestinal cancer were included in the 35 studies. According to the intervention period, 21 preoperative groups, 11 postoperative groups, and 12 perioperative groups. According to the type of control, 26 groups were on a standard diet, and 18 were no supplement. Seven of the tumor types were gastric cancers (24, 26, 28, 43–46), seven were colorectal cancers (47–52, 59), five were esophageal cancers (25, 29, 53, 54, 58), three were periampullary cancers (including pancreatic cancer) (22, 56, 57), and others were mixed types (23, 27, 30, 34–42, 55). In addition, malnutrition rates before intervention were reported in 20 of the 35 studies, with all participants well-nourished in four studies (25, 37, 42, 48), all participants malnourished in four studies (34, 40, 41, 55), and patients in the remaining 12 studies were mixed (22, 24, 28–30, 35, 39, 45, 47, 49, 56, 58).

**Table 1:** Characteristics of included clinical trials in the meta-analysis.

**References**	**Country**	**Total size**	**Tumor types**	**Time of administration**	**Duration of intervention (days)**	**Enteral immunonutrition composition**	**Control^a^**
Aida et al. (56)	Japan	50	Periampullary cancer	Preoperative	5	Arginine, ω-3 fatty acids, RNA	No supplement
Braga et al. (34)	Italy	150	Gastrointestinal cancer	Preoperative	7	Arginine, ω-3 fatty acids, RNA	No supplement
				Perioperative	14		
Braga et al. (49)	Italy	200	Colorectal cancer	Perioperative	>5	Arginine, ω-3 fatty acids, RNA	No supplement
				Preoperative	5		Standard diet
							No supplement
Farreras et al. (45)	Spain	60	Gastric cancer	Postoperative	7	Arginine, ω-3 fatty acids, RNA	Standard diet
Fujitani et al. (28)	Japan	231	Gastric cancer	Preoperative	5	Arginine, ω-3 fatty acids, RNA	No supplement
Gade et al. (57)	Denmark	35	Pancreatic cancer	Preoperative	7	Arginine, ω-3 fatty acids, RNA	No supplement
Gianotti et al. (37)	Italy	305	Gastrointestinal cancer	Preoperative	5	Arginine, ω-3 fatty acids, RNA	No supplement
				Perioperative	>5		
Giger et al. (35)	Switzerland	29	Gastrointestinal cancer	Preoperative	5	Arginine, ω-3 fatty acids, RNA	No supplement
Giger-Pabst et al. (42)	Switzerland	108	Gastrointestinal cancer	Preoperative	3	Arginine, ω-3 fatty acids, RNA	Standard diet
Gunerhan et al. (55)	Turkey	33	Gastrointestinal cancer	Preoperative	7	Arginine, ω-3 fatty acids, RNA	Standard diet
							No supplement
Hamza et al. (22)	England	30	Periampullary cancer	Perioperative	21	Arginine, ω-3 fatty acids, RNA	Standard diet
Horie et al. (48)	Japan	67	Colorectal cancer	Preoperative	5	Arginine, ω-3 fatty acids, RNA	No supplement
Kanekiyo et al. (25)	Japan	40	Esophageal cancer	Perioperative	14	Arginine, ω-3 fatty acids, RNA	Standard diet
Kitagawa et al. (58)	Japan	29	Esophageal cancer	Preoperative	5	Arginine, ω-3 fatty acids, RNA, glutamine	Standard diet
Klek et al. (39)	Poland	183	Gastrointestinal cancer	Postoperative	7	Arginine, ω-3 fatty acids, glutamine	Standard diet
Klek et al. (30)	Poland	105	Gastrointestinal cancer	Postoperative	7	Arginine, ω-3 fatty acids, glutamine	Standard diet
Klek et al. (40)	Poland	305	Gastrointestinal cancer	Postoperative	7	Arginine, ω-3 fatty acids, glutamine	Standard diet
Klek et al. (41)	Poland	84	Gastrointestinal cancer	Postoperative	7	Arginine, ω-3 fatty acids, glutamine	Standard diet
Lee et al. (47)	Korea	161	Colorectal cancer	Preoperative	7	Arginine, ω-3 fatty acids	No supplement
Li et al. (54)	China	103	Esophageal cancer	Perioperative	14	Arginine, ω-3 fatty acids, RNA, glutamine	Standard diet
Liu et al. (46)	China	78	Gastric cancer	Postoperative	7	Arginine, glutamine	Standard diet
							No supplement
Lobo et al. (36)	England	108	Gastrointestinal cancer	Postoperative	10-15	Arginine, ω-3 fatty acids, glutamine	Standard diet
Ma et al. (43)	China	34	Gastric cancer or GIST	Perioperative	5-16	Arginine, ω-3 fatty acids, glutamine	Standard diet
Marano et al. (24)	Italy	109	Gastric cancer	Postoperative	7	Arginine, ω-3 fatty acids, RNA	Standard diet
Moriya et al. (59)	Japan	85	Colorectal cancer	Preoperative	5	Arginine, ω-3 fatty acids, RNA	No supplement
Moya et al. (50)	Spain	122	Colorectal cancer	Perioperative	12	Arginine, ω-3 fatty acids, RNA	No supplement
Moya et al. (51)	Spain	244	Colorectal cancer	Perioperative	12	Arginine, ω-3 fatty acids, RNA	Standard diet
Mudge et al. (29)	Australia	263	Esophageal cancer	Preoperative	7	Arginine, ω-3 fatty acids, RNA	Standard diet
				Postoperative			
				Perioperative	14		
Nakamura et al. (38)	Japan	26	Gastrointestinal cancer	Preoperative	5	Arginine, ω-3 fatty acids, RNA	No supplement
Okamoto et al. (44)	Japan	60	Gastric cancer	Preoperative	7	Arginine, ω-3 fatty acids, RNA	Standard diet
Sakurai et al. (53)	Japan	30	Esophageal cancer	Perioperative	6	Arginine, ω-3 fatty acids, RNA	Standard diet
Scislo et al. (26)	Poland	98	Gastric cancer	Postoperative	6	Arginine, ω-3 fatty acids, glutamine	Standard diet
Wierdak et al. (52)	Poland	26	Colorectal cancer	Preoperative	14	Arginine, ω-3 fatty acids, RNA	Standard diet
Xu et al. (23)	China	60	Gastrointestinal cancer	Preoperative	7	Arginine, ω-3 fatty acids, RNA	Standard diet
Yildiz et al. (27)	Turkey	41	Gastrointestinal cancer	Perioperative	14	Arginine, glutamine	Standard diet

a *Standard diet refers to an isocaloric and isonitrogenous enteral nutrition supplement and no supplement refers to a normal diet without supplements*.

*RNA, ribonucleic acid; GIST, gastrointestinal stromal tumor*.

Overall complications and infectious complications were the primary outcome measures. Non-infectious complications, mortality, length of hospital stay, and enteral nutrition-related complications were the secondary outcome measures.

The quality of each study was appraised through the Cochrane Collaboration's tool. Supplementary Figures 1, 2 present the quality assessment of studies.

### Results of Meta-Analysis

**Figure 2:**
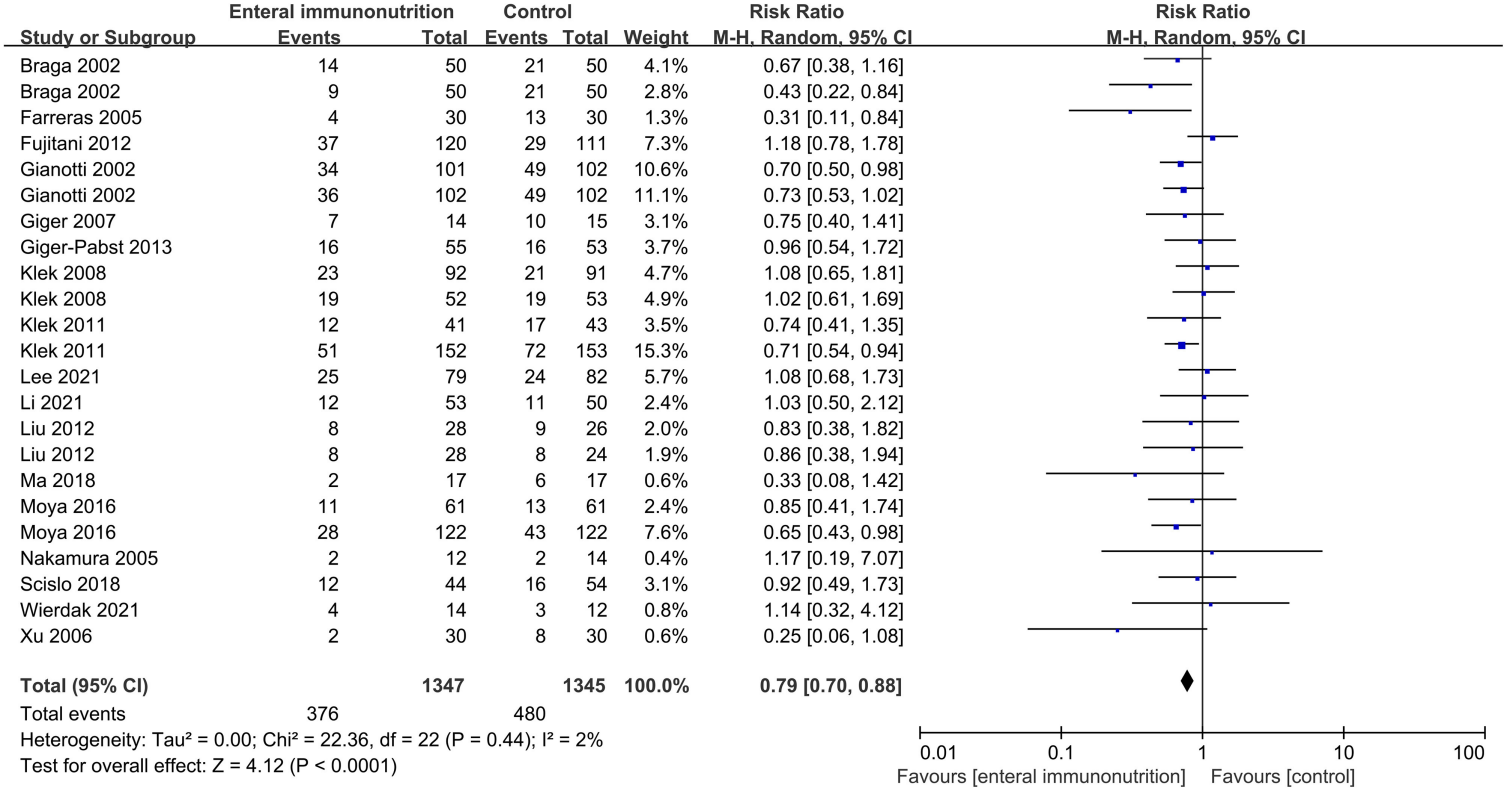
Forest plot of meta-analysis of overall complications.

**Figure 3:**
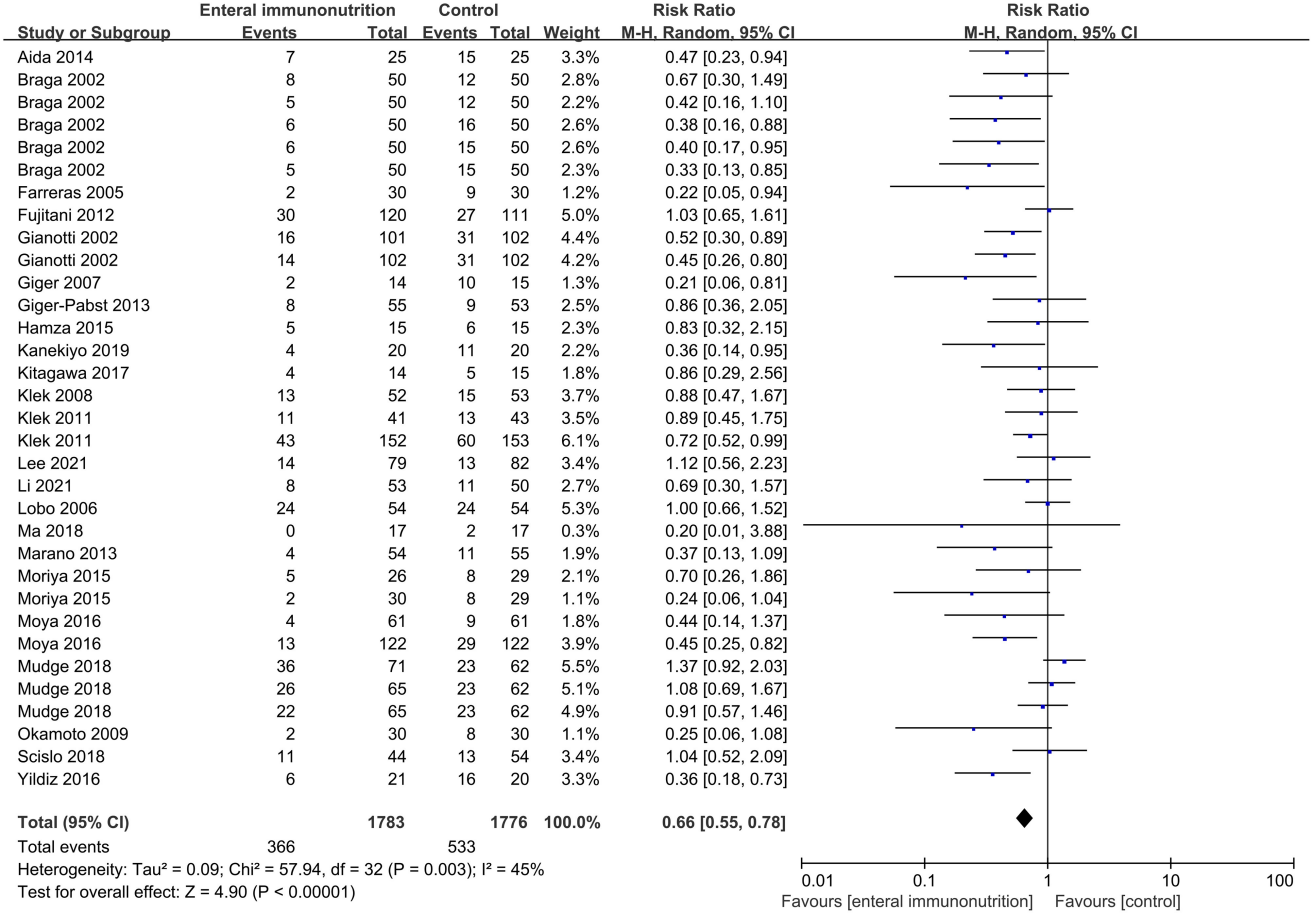
Forest plot of meta-analysis of infectious complications.

In this study, all 20 studies provided relevant data for the overall complications of 1,347 patients in EIN group vs. 1,345 patients in the control group (23, 28, 30, 34, 35, 37–43, 45, 46, 55). Compared with the control group, EIN group had a significantly decreased incidence of overall complications, and the pooled RR was 0.79 (95% CI: 0.70–0.88; *p* < 0.001; I^2^ = 2%; Figure 2). Then, the subgroup analyses of infectious, non-infectious, length of hospital stay, mortality, and enteral nutrition-related were performed. Among 26 studies that reported the data (24, 27, 30, 34–37, 40–45, 55, 59), the incidence of infectious complications was significantly lower in patients with EIN administration than in the control group, and the pooled RR was 0.66 (95% CI: 0.55–0.78; *p* < 0.001; I^2^ = 45%; Figure 3). When compared to patients in the control group, surgical site infection, abdominal abscess, anastomotic leakage, bacteremia, duration of systemic inflammatory response syndrome (SIRS), and duration of antibiotic therapy were significantly lower in the specific infectious complications treated with EIN administration. However, there was no significant difference between the two groups with other infectious complications, such as respiratory tract infection, urinary tract infection, and respiratory failure. Furthermore, when compared to the control group, EIN group had a significantly shorter length of hospital stay. Still, there was no significant effect of EIN on non-infectious complications or mortality. There was no significant difference in enteral nutrition-related complications between the two groups. Table 2 contains more specific information.

**Table 2:** Analysis of enteral immunonutrition outcomes.

**Enteral immunonutrition vs. control**	**No. of studies**	**RR**	**95%CI**	***p***	**Heterogeneity (I^**2**^)**
**Infectious**					
Surgical site infection	28	0.66	0.53, 0.83	<0.001	11%
Respiratory tract infection	29	0.88	0.75, 1.04	0.14	0%
Urinary tract infection	17	0.71	0.49, 1.02	0.06	0%
Respiratory failure	8	0.96	0.61, 1.53	0.88	0%
Abdominal abscess	18	0.60	0.41, 0.86	0.005	0%
Infection of venous catheter	7	0.71	0.32, 1.56	0.39	0%
Pancreatic fistula	9	0.89	0.58, 1.35	0.57	0%
Duodenal fistula	4	1.24	0.38, 3.97	0.72	0%
Anastomotic leakage	18	0.65	0.49, 0.85	0.002	0%
Bacteremia	6	0.35	0.19, 0.64	<0.001	0%
Sepsis	12	0.68	0.41, 1.11	0.12	0%
SIRS	3	1.20	0.84, 1.69	0.31	0%
Duration of SIRS	4	−0.35^*^	−0.48, −0.23	<0.001	85%
Duration of antibiotic therapy	4	−2.50^*^	−3.11, −1.88	<0.001	63%
**Non-infectious**					
Non-infectious complications	15	0.91	0.80, 1.02	0.10	0%
Vein thrombosis	5	0.70	0.21, 2.38	0.57	0%
Pulmonary thrombosis	4	0.54	0.13, 2.26	0.40	0%
Arrythmia	4	0.81	0.38, 1.71	0.58	0%
Myocardial infarction	3	2.97	0.47, 18.65	0.25	0%
Cardiac dysfunction	7	0.72	0.28, 1.84	0.49	0%
Renal dysfunction	7	1.27	0.56, 2.92	0.57	0%
Delayed gastric emptying	7	0.95	0.60, 1.51	0.83	0%
Intestinal obstruction	14	0.89	0.57, 1.38	0.60	0%
Wound dehiscence	10	0.65	0.34, 1.22	0.18	0%
Postoperative bleeding	13	0.68	0.37, 1.25	0.21	0%
Pleural effusion	4	0.74	0.36, 1.55	0.43	0%
Length of hospital stay	20	−2.03^*^	−2.97, −1.10	<0.001	82%
Mortality	16	0.67	0.40, 1.11	0.12	0%
**Enteral nutrition related**					
Adverse effects	7	0.91	0.73, 1.14	0.42	0%
Bloating	4	0.85	0.48, 1.49	0.57	0%
Vomiting	5	1.23	0.66, 2.29	0.51	0%
Diarrhea	9	0.81	0.57, 1.16	0.25	0%

* *indicates continuous data, using mean difference*.

*RR, risk ratio; CI, confidence interval; SIRS, systemic inflammatory response syndrome*.

### Analysis of Specific Cancer

#### Gastric Cancer

Supplementary Table 2 presents the results of gastric cancer. Seven articles (24, 26, 28, 43–46), including 670 patients, reported information related to gastric cancer. The incidence of overall complications, non-infectious, length of hospital stay, mortality, and enteral nutrition-related complications had no significant statistical difference between the two groups. SIRS duration was significantly reduced when compared to the control group when EIN was administered, but other infectious complications did not differ significantly between the two groups.

#### Colorectal Cancer

The outcomes of colorectal cancer are presented in Supplementary Table 3. The incidence of overall complications and non-infectious between the experimental and control group represented a non-significant difference. When compared to the control, EIN administration resulted in a significant reduction in the length of hospital stay. In the infectious subgroup, compared to the control, EIN administration reduced the incidence of infectious complications and surgical site infection statistically significantly, but no significant effects were seen for other infectious complications.

#### Esophageal Cancer

There was no significant difference in postoperative outcomes, including infectious and length of hospital stay, between EIN administration and controls in esophageal cancer patients. The details are presented in Supplementary Table 4.

#### Periampullary Cancer (Including Pancreatic Cancer)

The results of periampullary cancer (including pancreatic cancer) are presented in Supplementary Table 5. In the infectious subgroup, compared to the control, the incidence of infectious complications and surgical site infection was significantly lower in patients with EIN, but there was no difference between the two groups for other infectious complications. There was no significant difference in non-infectious complications between the two groups.

### Analysis of Different Intervention Periods

#### Preoperative

Supplementary Table 6 shows the results of preoperative nutrition. In terms of overall complications, non-infectious complications, mortality, and enteral nutrition-related complications, EIN administration had no significant effect compared to the control. Preoperative EIN administration significantly reduced the incidence of infectious complications, anastomotic leakage, bacteremia, duration of SIRS, and duration of antibiotic therapy compared to the control, but other infectious complications showed no significant difference between the two groups. Moreover, the length of hospital stay was significantly shortened in the experimental group compared with the control group.

#### Postoperative

The outcomes of postoperative nutrition are presented in Supplementary Table 7. The incidence of overall complications was significantly lower with EIN administration compared with the control, and the pooled RR was 0.80 (95% CI: 0.66–0.96; *p* = 0.02; I^2^ = 0%). There was no significant difference between the experimental and control groups in non-infectious, length of hospital stay, mortality, or enteral nutrition-related complications. When compared to the control, the incidence of surgical site infection and bacteremia was significantly lower with postoperative EIN administration, but other infectious complications showed no significant difference between the two groups.

#### Perioperative

The outcomes of perioperative nutrition are presented in Table 3. The incidence of overall complications was significantly reduced with EIN administration compared with the control, and the pooled RR was 0.68 (95% CI: 0.54–0.84; *p* < 0.001; I^2^ = 0%). There was a significant reduction in the incidence of infectious complications, surgical site infection, abdominal abscess, anastomotic leakage, bacteremia, and duration of antibiotic therapy with perioperative EIN administration compared to the control group, but no significant difference was demonstrated in other infectious complications between the two groups. In comparison to the control, there was no significant effect of EIN on non-infectious, mortality or enteral nutrition-related complications, but the length of hospital stay was significantly reduced.

**Table 3:** Analysis of perioperative nutrition outcomes.

**Enteral immunonutrition vs. control**	**No. of studies**	**RR**	**95%CI**	***p***	**Heterogeneity (I^**2**^)**
Overall complications	6	0.68	0.54, 0.84	<0.001	0%
**Infectious**
Infectious complications	11	0.54	0.37, 0.79	0.001	59%
Surgical site infection	10	0.47	0.31, 0.71	<0.001	0%
Respiratory tract infection	10	0.90	0.62, 1.31	0.57	0%
Urinary tract infection	6	0.55	0.24, 1.28	0.17	0%
Respiratory failure	5	1.07	0.51, 2.25	0.86	0%
Abdominal abscess	5	0.36	0.14, 0.90	0.03	0%
Infection of venous catheter	3	0.57	0.22, 1.47	0.25	0%
Pancreatic fistula	3	1.26	0.50, 3.18	0.62	0%
Anastomotic leakage	9	0.57	0.37, 0.90	0.02	0%
Bacteremia	3	0.24	0.06, 0.96	0.04	0%
Sepsis	4	0.85	0.38, 1.92	0.70	0%
Duration of antibiotic therapy	3	−2.80^*^	−3.79, −1.82	<0.001	50%
**Non-infectious**
Non-infectious complications	7	0.85	0.69, 1.04	0.11	0%
Arrythmia	2	1.00	0.27, 3.67	1.00	0%
Renal dysfunction	2	0.62	0.13, 3.04	0.56	0%
Delayed gastric emptying	4	0.85	0.38, 1.92	0.70	0%
Intestinal obstruction	5	0.74	0.41, 1.35	0.33	0%
Wound dehiscence	4	0.72	0.26, 1.99	0.52	0%
Postoperative bleeding	5	0.49	0.14, 1.67	0.25	0%
Pleural effusion	2	0.55	0.13, 2.31	0.41	0%
Length of hospital stay	6	−2.38^*^	−3.20, −1.56	<0.001	0%
Mortality	5	0.89	0.29, 2.75	0.84	0%
**Enteral nutrition related**
Adverse effects	3	1.00	0.74, 1.37	0.98	0%
Bloating	2	0.83	0.45, 1.53	0.54	0%
Vomiting	3	1.18	0.51, 2.70	0.70	0%
Diarrhea	4	1.24	0.62, 2.47	0.55	0%

* *indicates continuous data, using mean difference*.

*RR, risk ratio; CI, confidence interval*.

### Analysis of Control Groups

#### Standard Diet

Supplementary Table 8 presents the results of the standard diet as the control. The incidence of overall complications was significantly reduced in the experimental group compared with the control group, and the pooled RR was 0.78 (95% CI: 0.66–0.92; *p* = 0.003; I^2^ = 4%). In comparison to the control, there was no significant effect of EIN on non-infectious, mortality or enteral nutrition-related complications, but the length of hospital stay was significantly reduced. In the infectious subgroup, compared to the control, the incidence of infectious complications, surgical site infection, abdominal abscess, bacteremia, and duration of SIRS was significantly lower in patients with EIN, but other infectious complications were not significantly different between the two groups.

#### No Supplement

Supplementary Table 9 presents the results of no supplement as the control. Compared with the control, there was a significant reduction with EIN administration in the incidence of overall complications, and the pooled RR was 0.80 (95% CI: 0.67–0.94; *p* = 0.009; I^2^ = 8%). In comparison to the control, there was no significant effect of EIN on non-infectious, mortality or enteral nutrition-related complications, but the length of hospital stay was significantly reduced. In the infectious subgroup, compared to the control, the incidence of infectious complications, surgical site infection, abdominal abscess, anastomotic leakage, bacteremia, duration of SIRS, and duration of antibiotic therapy was significantly lower in EIN patients, but other infectious complications were not significantly different between the two groups.

### Analysis of Nutriture

#### Malnourished

Supplementary Table 10 presents the results of malnourished patients. All four studies (34, 40, 41, 55), including 572 participants, provided relevant data for malnourished patients. The incidence of overall complications in malnourished patients showed a significant reduction in EIN group vs. the control group, and the pooled RR was 0.67 (95% CI: 0.54–0.84; *p* < 0.001; I^2^ = 0%). In EIN group, there was a significant reduction in the incidence of infectious complications and bacteremia when compared to the control group, but there was no significant difference between the two groups for other infectious complications. There was no significant effect of EIN on non-infectious complications when compared to the control, but the length of hospital stay and mortality were significantly lower.

#### Well-Nourished

Supplementary Table 11 presents the results of well-nourished patients. All four studies (25, 37, 42, 48), including 520 participants, provided relevant data for malnourished patients. The incidence of overall complications in well-nourished patients showed a significant reduction in EIN group vs. the control group, and the pooled RR was 0.75 (95% CI: 0.60–0.93; *p* = 0.01; I^2^ = 0%). Compared with the control, no significant effect of EIN was seen for non-infectious, mortality, and enteral nutrition-related complications, but the length of hospital stay was significantly shortened. Compared with the control, there was a significant reduction in the incidence of infectious complications, surgical site infection, abdominal abscess, and anastomotic leakage in EIN group, but other infectious complications showed no significant difference between the two groups.

### Sensitivity Analysis and Publication Bias

The funnel charts for the studies reporting overall complications and infectious compilations were roughly symmetrical, indicating that no studies had a significant publication bias (Supplementary Figures 3, 4). Sensitivity analysis revealed that the outcomes of all studies were consistent.

## Discussion

Patients with gastrointestinal cancers often suffer from malnutrition (2, 3), which is associated with impaired cellular and humoral immune function and changes in inflammatory responses (12–18). Therefore, perioperative nutritional support is critical. However, the benefits of EIN in terms of clinical outcomes and immune markers remain debatable. Given this, we conducted a systematic review and meta-analysis. This meta-analysis included patients undergoing surgery for different gastrointestinal cancers, primarily gastric, colorectal, esophageal, periampullary (including pancreatic), and mixed types. The EIN was defined as containing at least two or more nutrients, including arginine, glutamine, omega-3 fatty acids, and nucleotides. In the included studies, most of the nutritional formulations were a combination of arginine, ω-3 fatty acids, glutamine; some were a combination of arginine, ω-3 fatty acids, glutamine; and a few were arginine, glutamine, or arginine, ω-3 fatty acids, RNA, glutamine, or arginine, ω-3 fatty acids. The duration of administration ranges from a minimum of 3 days to a maximum of 21 days. The dose of EIN also differed.

The results of this systematic review and meta-analysis showed that, in patients undergoing surgery for gastrointestinal cancer, compared with standard diet or no supplement, EIN administration effectively reduced the incidence of overall complications, infectious complications, and length of hospital stay, but not in reducing the incidence of non-infectious complications or mortality. Moreover, the incidence of enteral nutrition-related complications had no significant association with EIN administration. In infectious complications, EIN could reduce the risk of surgical site infection, abdominal abscess, anastomotic leakage, bacteremia, duration of SIRS, and duration of antibiotic therapy. However, EIN's effects on infectious complications were limited. EIN was not associated with the incidence of respiratory tract infection, urinary tract infection, respiratory failure, infection of the venous catheter, pancreatic fistula, duodenal fistula, sepsis, and SIRS.

The intestinal tract has both physiological and immune barriers (60). The physiological barrier is formed by the tight junctions between the epithelial cells and the epithelial cells (60). Gut-associated lymphoid tissue, which includes peyer patches, intraepithelial lymphocytes, and lamina propria lymphocytes, functions as an immune barrier in the intestine (60). Surgery can damage the defense mechanism, change the intestinal flora, and lead to various postoperative complications (10, 11, 61).

To a certain extent, EIN can reduce the occurrence of infectious complications. This could be because EIN boosts immune response and reduces inflammation in gastrointestinal surgery. Both Li and Chen confirmed that CD4 cell counts and the CD4/CD8 ratio were eventually higher in EIN group compared to the control group in gastric cancer patients undergoing gastrectomy (21, 62). Concurrently, TNF-α levels were significantly lower (21, 62). Additionally, specific nutrients in EIN play their respective roles in immune response and anti-infection. Arginine therapy could markedly increase intestinal IgA levels, stimulate lymphocyte function, and improve wound healing (63–65). Glutamine is essential for cellular immunity, maintaining gut barrier function, and synthesizing the endogenous antioxidant glutathione (63, 64, 66). Omega-3 fatty acids reduce responsiveness to cytokines and the systemic inflammatory response by affecting membrane phospholipids composition to produce the lipid mediators with lower bio-activity, stabilize NFkB/IkB complex, and act as agonists for peroxisomal proliferators-activated receptors (63, 64). Ribonucleic acid (RNA) can stimulate T lymphocytes' maturation and phenotypic expression (66). In short, EIN is primarily composed of arginine, glutamine, omega-3 fatty acids, and nucleotides, and it has the potential to reduce overall complications, particularly infectious complications, *via* several pathways.

Anastomotic leakage, one of the most severe complications in gastrointestinal surgery, is associated with a prolonged hospital stay and increased risk of morbidity and mortality (67–75). Normal anastomosis healing is divided into four stages: hemostasis, inflammation, proliferative, and remodeling. Numerous gastrointestinal aerobic and anaerobic bacteria and the role of increased loads of collagenases and matrix metalloproteinase will lead to the occurrence of infectious complications during the anastomosis healing process (71). Besides, malnutrition is a significant risk factor for developing anastomotic leakage (76–79). Thus, appropriate nutritional support is essential to prevent anastomotic leakage. Therefore, on the one hand, EIN contributes to improving the nutritional status of patients; on the other hand, EIN conduces to maintain the gut-associated lymphoid tissue function, stimulates tissue growth after infection, and thus modulates dysfunction of the intestinal barrier, promotes wound healing, and achieves the effect of reducing anastomotic leakage (63, 64, 66, 80). Yildiz et al. found that EIN reduced the incidence of anastomotic leakage undergoing gastrointestinal surgery (27). Our meta-analysis reached a consistent conclusion.

Developing surgical site infection involves many factors such as microbial characteristics, patient characteristics, and surgical characteristics (81). Surgical site infection is mainly caused by endogenous infection (81), among which anastomotic leakage is a crucial cause of surgical site infection (82). During anastomotic leakage, abscess formation and septic complications caused by intraperitoneal spillage of feculent material and considerable bowel leakage could cause the direct or hematogenous spread of the infected surgical site (82). Our study found that EIN reduced the incidence of surgical site infection, probably because EIN can maintain the number of gut-associated lymphoid tissue cells and IgA levels in the intestinal lumen, thus maintaining the intestinal immune barrier and preventing the transfer of bacteria from the intestinal tract, playing a role in fighting infection to some extent (60, 66).

The abdominal abscess may be secondary to anastomosis leaks or be caused by a distant blood spread of infection (83). For example, abdominal abscess after pancreaticoduodenectomy is likely the consequence of pancreatic fistula or leakage (84). Developing abdominal abscesses depends on bacterial contamination, the virulence of the bacteria, and the patient's resistance and defense system (83). In our study, EIN administration significantly reduced the incidence of the abdominal abscess. EIN may play a role in preserving the intestinal mucosal barrier, preventing bacteria from spreading, and boosting the immune system (66).

In patients undergoing surgery for gastrointestinal cancer, compared with a standard diet or no supplement, EIN cannot reduce the incidence of any non-infectious complications included in this study, such as pulmonary thrombosis, vein thrombosis, delayed gastric emptying, and intestinal obstruction. Although EIN can maintain some intestinal function, postoperative intestinal peristalsis is influenced by various factors. Postoperative peritoneal irritation or inflammation causes sympathetic nerve excitation, inhibiting gastrointestinal motility (85–88). In addition, the release of cytokines and other inflammatory mediators during inflammation reduces gastrointestinal motility (85–88). Another critical factor is the use of opioids. Opioids act upon μ-opioid receptors in the myenteric and submucosal neurons in the gut (85–88). These elements can cause intestinal obstruction and delayed gastric emptying. Furthermore, EIN was not linked to thrombosis. Three factors contribute to venous thrombosis: vein damage, blood stasis, and hypercoagulability (89). In the surgical setting, venous stasis is considered one of the significant triggers of thrombosis (90, 91). Prolonged operative time and general anesthesia-induced vasodilation lead to potential venous stasis, which induces pulmonary thrombosis and vein thrombosis (91). However, Zhang et al.'s meta-analysis demonstrated that perioperative EIN reduced postoperative non-infectious complications in patients undergoing gastrointestinal cancer surgery, which may be due to perioperative EIN could ameliorate splanchnic microperfusion and oxygenation and increase immune response (92).

EIN administration was not associated with an increase in the incidence of enteral nutrition-related complications, indicating that EIN was well tolerated. Our study discovered that EIN could reduce the length of hospital stay in gastrointestinal cancer patients undergoing surgery, most likely due to EIN's ability to reduce the occurrence of anastomotic leakage, surgical site infection, and other complications, which may be risk factors for length of hospital stay (93). In addition, EIN improves patients' nutrition to prevent the prolonged length of hospital stay. Nevertheless, EIN did not reduce mortality. Our results are consistent with Wong et al. Their meta-analysis demonstrated that EIN reduced the length of hospital stay but cannot reduce the incidence of mortality in patients undergoing upper gastrointestinal surgery (94). Various factors, such as characteristics of the disease, the patient's preoperative condition, operation type, and postoperative complications, are associated with mortality after gastrointestinal surgery (95, 96). In addition, EIN's anti-infection effect is also limited, so it is challenging to decrease postoperative mortality across a single measure.

EIN appeared to be more effective in patients with colorectal cancer in analyzing specific cancers. When compared to the control, EIN significantly reduced the incidence of infectious complications, surgical site infection, and length of hospital stay in colorectal cancer. Intestinal bacteria reside mainly in the lower gastrointestinal tract (97), and infectious complications of the lower gastrointestinal tract have a relatively high incidence (98, 99), so EIN may have a more significant improvement effect on postoperative infection for colorectal cancer. Moreover, the inadequate sample size in subgroups and variation in amount and duration of EIN administration could contribute to this. Further studies are required in the subgroup of specific cancers. In the analysis of the intervention period, perioperative EIN outperformed preoperative or postoperative in reducing the incidence of infections and could also shorter the length of hospital stay. This is consistent with the conclusion of Song et al. (100), which further confirmed that perioperative EIN administration is the optimum option for patients undergoing surgery for gastrointestinal cancer. When compared to the standard diet in the control group, EIN was more effective in reducing the incidence of postoperative complications when no supplement was used, implying the importance of nutrition supplements. When specific nutritional conditions were examined, EIN was found to reduce overall complications, some infectious complications, and length of hospital stay in well-nourished and malnourished patients compared to controls. It is worth noting that the mortality was significantly decreased in malnourished groups with EIN administration, which seems that EIN was more efficient for malnourished patients. Due to malnutrition being a significant risk factor for postoperative complications (101–103), EIN can significantly improve postoperative complications by improving the nutritional status of malnourished patients. The likely reason is that EIN helps malnourished patients reduce inflammation, accelerate wound healing, prevent severe complications, and thus reduce mortality (60). Nevertheless, most studies have failed to prove that EIN reduces mortality in surgical patients (94, 104, 105). Regrettably, we did not have sufficient data for further analysis of the effect of EIN in the malnourished and well-nourished group for postoperative complications. As a result, the impact of EIN on mortality remains to be further studied, and more randomized trials are warranted to focus on the effect of EIN on postoperative complications in people with different nutritional statuses.

## Strengths and Limitations

There are several limitations to the current systematic analysis that should be considered. First, this study includes unavoidable heterogeneity, such as variations in operation, disease severity, duration of intervention, and definition of complications. Second, some subgroup analyses used small sample sizes, which reduced the credibility of the results. Furthermore, some problems remain to be solved, such as the best formula, ratio, and amount of EIN and the influence of EIN on postoperative outcomes of patients with different types of gastrointestinal tumors. This systematic review and meta-analysis, on the other hand, thoroughly examined the effect of EIN on postoperative outcomes in patients undergoing surgery for gastrointestinal cancers, including subgroup analysis of specific tumor types, EIN administration period, control group type, and patient nutrition.

## Conclusion

According to this systematic review and meta-analysis, EIN is safe and beneficial for reducing overall complications, infectious complications, and length of hospital stay, but it has no efficacy for reducing non-infectious complications in patients undergoing surgery for gastrointestinal cancer (including gastric cancer, colorectal cancer, esophageal cancer, periampullary cancer, or pancreatic cancer). In terms of infectious complications, EIN primarily minimizes the incidence of surgical site infection, abdominal abscess, anastomotic leakage, bacteremia, SIRS duration, and antibiotic therapy duration. Therefore, perioperative EIN administration is recommended for malnourished patients undergoing surgery for gastrointestinal cancer, especially for patients with colorectal cancer. Overall, more well-designed and large-scale RCTs are required to clarify the unanswered questions and further evaluate the effect of EIN in patients undergoing gastrointestinal cancer surgery to provide reasonable theoretical guidelines for clinical practice.

## Data Availability Statement

The original contributions presented in the study are included in the article/Supplementary Material, further inquiries can be directed to the corresponding author/s.

## Author Contributions

JC designed the research process. JS and SD searched the database for corresponding articles and drafted the meta-analysis. ZL and WD extracted useful information from the articles above. JHo used statistical software for analysis. JHu polished this article. All authors had read and approved the manuscript and ensured that this was the case.

## Conflict of Interest

The authors declare that the research was conducted in the absence of any commercial or financial relationships that could be construed as a potential conflict of interest.

## Publisher's Note

All claims expressed in this article are solely those of the authors and do not necessarily represent those of their affiliated organizations, or those of the publisher, the editors and the reviewers. Any product that may be evaluated in this article, or claim that may be made by its manufacturer, is not guaranteed or endorsed by the publisher.

## Supplementary Material

Supplementary Figure 1 Risk of bias summary of studies included.

Supplementary Figure 2 Risk of bias graph of studies included.

Supplementary Figure 3 Funnel plot of meta-analysis of overall complications.

Supplementary Figure 4 Funnel plot of meta-analysis of infectious complications.

Supplementary Table 1 Characteristics of all studies included in the meta-analysis.

Supplementary Table 2 Analysis of gastric cancer outcomes.

Supplementary Table 3 Analysis of colorectal cancer outcomes.

Supplementary Table 4 Analysis of esophageal cancer outcomes.

Supplementary Table 5 Analysis of periampullary cancer (including pancreatic cancer) outcomes.

Supplementary Table 6 Analysis of preoperative nutrition outcomes.

Supplementary Table 7 Analysis of postoperative nutrition outcomes.

Supplementary Table 8 Analysis of standard diet as control groups.

Supplementary Table 9 Analysis of no supplement as control groups.

Supplementary Table 10 Analysis of malnourished patients outcomes.

Supplementary Table 11 Analysis of well-nourished patients outcomes.

## Abbreviations

EIN: enteral immunonutrition
RCT: randomized controlled trial
RR: risk ratio
CI: confidence interval
MD: mean difference
SIRS: systematic inflammatory response syndrome
RNA: ribonucleic acid.
