# Supplementary figures and images for: Effect of Enteral Immunonutrition in Patients Undergoing Surgery for Gastrointestinal Cancer: An Updated Systematic Review and Meta-Analysis
Source: Front Nutr. 2022 Jun 29;9:941975. doi: 10.3389/fnut.2022.941975 (PMC9277464; doi:10.3389/fnut.2022.941975)

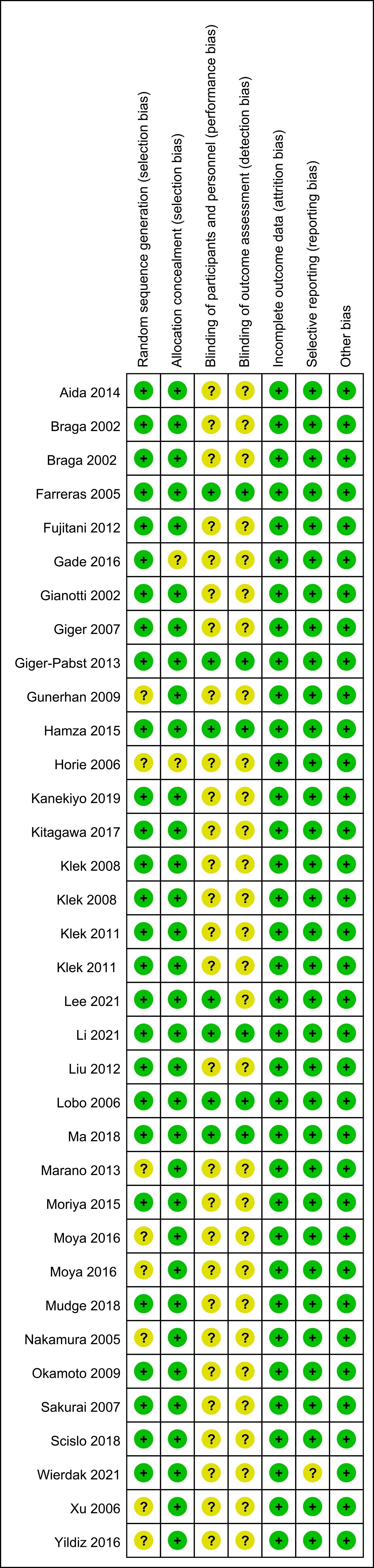

Supplement: Supplementary Figure 1 — Risk of bias summary of studies included. [file Image_1.TIF]

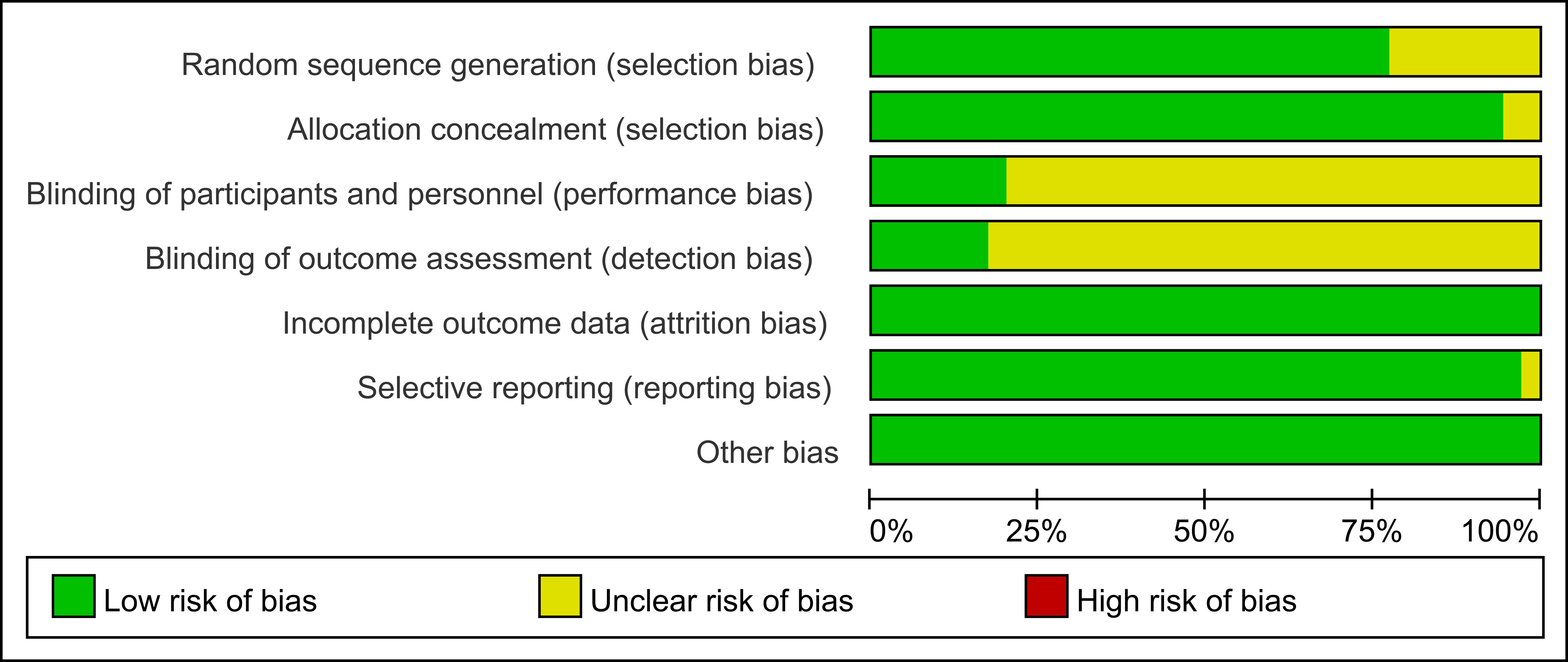

Supplement: Supplementary Figure 2 — Risk of bias graph of studies included. [file Image_2.TIF]

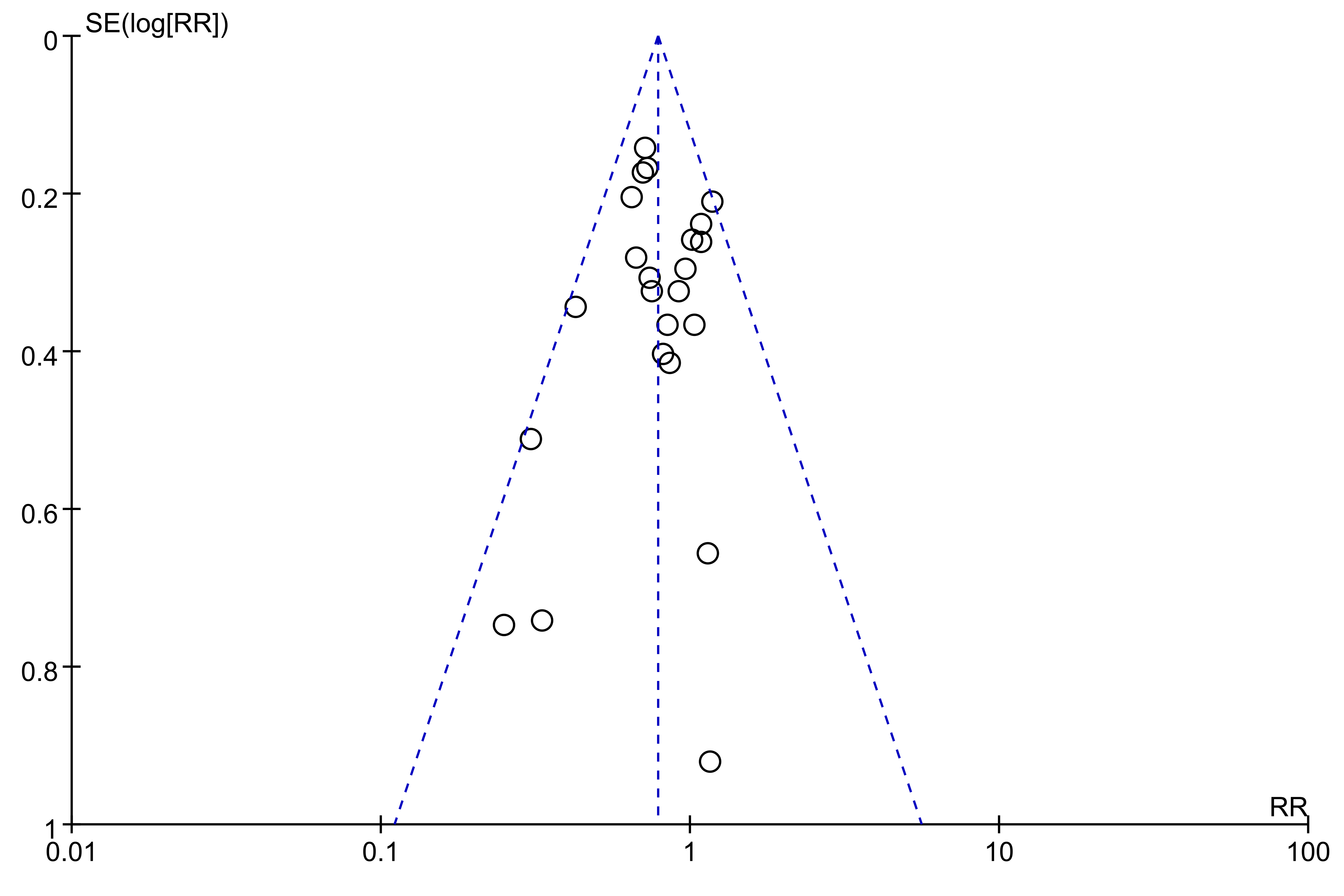

Supplement: Supplementary Figure 3 — Funnel plot of meta-analysis of overall complications. [file Image_3.TIF]

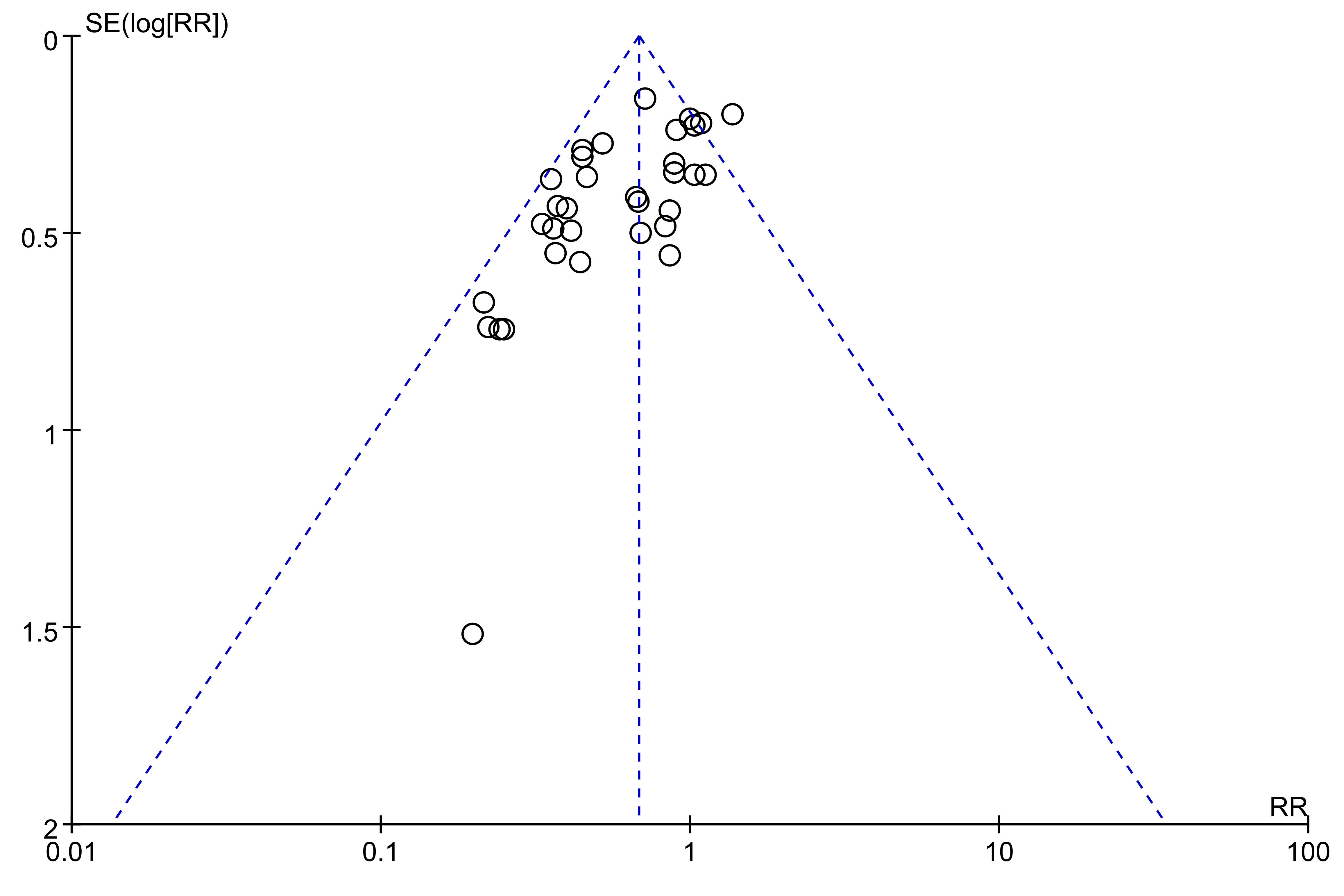

Supplement: Supplementary Figure 4 — Funnel plot of meta-analysis of infectious complications. [file Image_4.TIF]
