# Supplementary material for: Effect of Enteral Immunonutrition in Patients Undergoing Surgery for Gastrointestinal Cancer: An Updated Systematic Review and Meta-Analysis
Source: Front Nutr. 2022 Jun 29;9:941975. doi: 10.3389/fnut.2022.941975 (PMC9277464; doi:10.3389/fnut.2022.941975)
Supplement: Supplementary Table 1 — Characteristics of all studies included in the meta-analysis. [file Table_1.doc]

Supplementary Table 1-1. Characteristics of all studies included in the meta-analysis.

| Author, year | Recruitment time | Setting | Malnutrition rates  before intervention (%) | Definition of malnutrition | Feeding pattern | Target dose of enteral immunonutrition |
| --- | --- | --- | --- | --- | --- | --- |
| Aida, 2014 | 2009.5-2011.11 | Single-center | 8 | Weight loss more than 10% | Oral | 1000 kcal/d |
| Braga, 2002 | 1998.9-2000.12 | Single-center | 100 | Weight loss more than 10% | Oral, jejunal feeding | Pre: 1000 mL/d, post: 28 kcal/kg/d |
| Braga, 2002 | NR | NR | 10 | Weight loss more than 10% | Oral, jejunal feeding | Pre: 1000 mL/d, post: 1500 mL/d |
| Farreras, 2005 | 1999.1-2000.9 | Multicenter | 21.7 | Weight loss more than 10% | Jejunal feeding | Harris-Benedict formula |
| Fujitani, 2012 | NR | Multicenter | 2.2 | Weight loss more than 10% | Oral | 1000 mL/d |
| Gade, 2016 | 2012.3-2012.10 | Single-center | NR | NRS-2002 | Oral | 1.5 g protein/kg body weight |
| Gianotti, 2002 | 1998.10-2000.9 | Single-center | 0 | Weight loss more than 10% | Oral, jejunal feeding | Pre: 1000 mL/d, Post: 1500 mL/d |
| Giger, 2007 | 2001.1-2001.12 | Single-center | 62.1 | Weight loss more than 10% | Oral | 1000 mL/d |
| Giger-Pabst, 2013 | 2006.1-2008.5 | Multicenter | 0 | NRS-2002 | Oral | 750 mL/d |
| Gunerhan, 2009 | NR | Single-center | 100 | PG-SGA | Enteral feeding | Harris-Benedict formula |
| Hamza, 2015 | NR | Multicenter | 56.7 | MUST | Oral, jejunal feeding | Pre: 600 mL/d, post: 25 kcal/kg/d |
| Horie, 2006 | 2003.7-2004.7 | Single-center | 0 | NR | Oral | 750 mL/d |
| Kanekiyo, 2019 | NR | Single-center | 0 | NR | Oral, jejunal feeding | Pre: 750 mL/d, post: >10 ml/h |
| Kitagawa, 2017 | 2013.5-2015.3 | Single-center | 24.1 | Weight loss more than 10% | Oral | 600 mL/d |
| Klek, 2008 | 2004.6-2007.9 | Single-center | 18.6 | Weight loss more than 10% | Jejunal feeding | 100 mL/h |
| Klek, 2008 | 2001.6-2005.12 | Single-center | 16.2 | Weight loss more than 10% | Jejunal feeding | 100 mL/h |
| Klek, 2011 | 2003.1-2009.12 | Single-center | 100 | Weight loss more than 10% | Jejunal feeding | 100 mL/h |
| Klek, 2011 | 2001.6-2008.12 | Single-center | 100 | Weight loss more than 10% | Jejunal feeding | 100 mL/h |
| Lee, 2021 | 2019.4-2020.10 | Single-center | 8.7 | NRS-2002, MUST | Oral | 400 mL/d |
| Li, 2021 | 2017.12-2018.3 | Single-center | NR | NR | Oral, jejunal feeding | Pre: 500 mL/d, post: 23-30 kcal/kg/d |
| Liu, 2012 | 2005.8-2011.5 | Single-center | NR | NR | Jejunal feeding | 25-30 kcal/kg/d |
| Lobo, 2006 | 2000.1-2003.6 | Multicenter | NR | NR | Jejunal feeding | 75 mL/h |
| Ma, 2018 | 2011.6-2016.6 | Single-center | NR | NR | Oral, jejunal feeding | Pre: 400 mL/d, post: 1200 ml/d |
| Marano, 2013 | 2006-2011 | Single-center | 57.8 | NR | Jejunal feeding | 35 kcal/kg/d |
| Moriya, 2015 | 2002.10-2005.10 | Single-center | NR | NR | Oral | High: 750 mL/d |
|  |  |  |  |  |  | Low: 250 mL/d |
| Moya, 2016 | NR | Single-center | NR | NR | Oral | 800 mL/d |
| Moya, 2016 | 2014.1-2015.3 | Multicenter | NR | NR | Oral | 800 mL/d |
| Mudge, 2018 | 2009.11-2014.10 | Multicenter | 16.4 | PG-SGA | Oral, jejunal feeding | Pre: 900 mL/d, post: >40 mL/h |
| Nakamura, 2005 | NR | NR | NR | NR | Oral | 1000 mL/d |
| Okamoto, 2009 | 2005.4-2007.7 | Multicenter | NR | NR | Oral | 750 mL/d |
| Sakurai, 2007 | NR | Single-center | NR | NR | Oral, jejunal feeding | Pre: 1000 kcal/d, post: Harris-Benedict formula |
| Scislo, 2018 | 2004-2006 | Single-center | NR | Weight loss more than 10% | Jejunal feeding | 90-100 ml/h |
| Wierdak, 2021 | 2017.11-2018.11 | Single-center | NR | NR | Oral | Two servings per day |
| Xu, 2006 | 2003.1-2003.12 | Single-center | NR | NR | Nasal feeding | 25 Kcal/kg/d |
| Yildiz, 2016 | 2012.1-2013.2 | Single-center | NR | PG-SGA | Oral, jejunal feeding | Pre: 500 mL/d, post: <30 kcal/kg/d |

Pre, preoperative; Post, postoperative; NR, not reported; NRS-2002, Nutritional Risk Screening-2002; PG-SGA, Patient-Generated Subjective Global Assessment; MUST, Malnutrition Universal Screening Tool.

Supplementary Table 1-2. Characteristics of all the studies included in the meta-analysis.

| Author, year | Number of inclusions | | Mean age (years) | | BMI (kg/m^2^) | | Body weight (kg) | | Weight loss (%) | | Operation time (minutes) | | Transfused patients | |
| --- | --- | --- | --- | --- | --- | --- | --- | --- | --- | --- | --- | --- | --- | --- |
|  | Treatment | Control | Treatment | Control | Treatment | Control | Treatment | Control | Treatment | Control | Treatment | Control | Treatment | Control |
| Aida, 2014 | 25 | 25 | 66.4 | 65.1 | 21.5 | 22.6 | NR | NR | NR | NR | 494 | 491 | 4 | 3 |
| Braga, 2002 | 50 | 50 | 65.9 | 65.5 | NR | NR | 60.2 | 59.8 | 12.9 | 13.1 | 258 | 244 | 16 | 17 |
|  | 50 | 50 | 64.1 | 65.5 | NR | NR | 58.7 | 59.8 | 13.3 | 13.1 | 263 | 244 | 18 | 17 |
| Braga, 2002 | 50 | 50 | 60.5 | 62.2 | NR | NR | NR | NR | NR | NR | 190 | 197 | 11 | 9 |
|  | 50 | 50 | 63.0 | 61.8 | NR | NR | NR | NR | NR | NR | 202 | 188 | 9 | 8 |
|  | 50 | 50 | 63.0 | 62.2 | NR | NR | NR | NR | NR | NR | 202 | 197 | 9 | 9 |
| Farreras, 2005 | 30 | 30 | 66.7 | 69.2 | NR | NR | 66.4 | 62.4 | NR | NR | NR | NR | NR | NR |
| Fujitani, 2012 | 120 | 111 | 64.0 | 65.0 | 22.8 | 22.6 | NR | NR | 0 | 0 | NR | NR | NR | NR |
| Gade, 2016 | 19 | 16 | 68.0 | 69.0 | 24.3 | 23.8 | 70.5 | 70.5 | 5.5 | 7.9 | 275 | 297 | NR | NR |
| Gianotti, 2002 | 102 | 102 | 62.3 | 63.4 | 24.5 | 23.8 | 69.4 | 68.1 | 2.4 | 2.3 | 226 | 220 | 34 | 32 |
|  | 101 | 102 | 65.6 | 63.4 | 24.2 | 23.8 | 69 | 68.1 | 2.5 | 2.3 | 237 | 220 | 37 | 32 |
| Giger, 2007 | 14 | 15 | 64.4 | 63.0 | 23.7 | 22.7 | NR | NR | NR | NR | 388 | 400 | 8 | 9 |
| Giger-Pabst, 2013 | 55 | 53 | 64.9 | 63.2 | NR | NR | 77.5 | 73.2 | NR | NR | 235 | 241 | 9 | 11 |
| Gunerhan, 2009 | 13 | 11 | 64.6 | 61.3 | 24.1 | 22.2 | NR | NR | NR | NR | 304 | 289 | NR | NR |
|  | 13 | 9 | 64.6 | 64.4 | 24.1 | 23.0 | NR | NR | NR | NR | 304 | 249 | NR | NR |
| Hamza, 2015 | 15 | 15 | 63.0 | 67.0 | NR | NR | NR | NR | 9.2 | 9.6 | 413 | 364 | 8 | 7 |
| Horie, 2006 | 33 | 34 | 69.0 | 63.0 | 22.8 | 22.8 | 59 | 58.0 | NR | NR | 236 | 244 | NR | NR |
| Kanekiyo, 2019 | 20 | 20 | 65.0 | 60.0 | 21.9 | 21.5 | NR | NR | NR | NR | 435 | 419 | NR | NR |
| Kitagawa, 2017 | 14 | 15 | 67.1 | 66.8 | 21.0 | 19.9 | NR | NR | NR | NR | 573 | 595 | NR | NR |
| Klek, 2008 | 92 | 91 | 62.3 | 62.1 | NR | NR | NR | NR | NR | NR | NR | NR | NR | NR |
| Klek, 2008 | 52 | 53 | 61.2 | 61.4 | NR | NR | NR | NR | NR | NR | 311 | 298 | 12 | 12 |
| Klek, 2011 | 152 | 153 | 60.2 | 61.5 | 17.9 | 17.9 | NR | NR | 18.3 | 18.8 | NR | NR | NR | NR |
| Klek, 2011 | 41 | 43 | 65.0 | 61.0 | 17.0 | 17.0 | NR | NR | 17.0 | 19.0 | NR | NR | NR | NR |
| Lee, 2021 | 79 | 82 | 65.3 | 65.3 | 24.4 | 24.1 | NR | NR | NR | NR | NR | NR | NR | NR |
| Li, 2021 | 53 | 50 | 62.1 | 61.5 | 23.6 | 23.7 | NR | NR | NR | NR | 215 | 209 | NR | NR |
| Liu, 2012 | 28 | 24 | 57.3 | 58.4 | NR | NR | NR | NR | NR | NR | NR | NR | NR | NR |
|  | 28 | 26 | 57.3 | 56.2 | NR | NR | NR | NR | NR | NR | NR | NR | NR | NR |
| Lobo, 2006 | 54 | 54 | 65.7 | 66.6 | NR | NR | NR | NR | NR | NR | NR | NR | NR | NR |
| Ma, 2018 | 17 | 17 | 60.2 | 62.0 | 24.2 | 23.0 | NR | NR | NR | NR | NR | NR | NR | NR |
| Marano, 2013 | 54 | 55 | 66.6 | 65.1 | 23.1 | 23.6 | NR | NR | NR | NR | 310 | 367 | NR | NR |
| Moriya, 2015 | 26 | 29 | 64.7 | 63.8 | 22.6 | 22.2 | 57.3 | 57.1 | NR | NR | 246 | 223 | NR | NR |
|  | 30 | 29 | 64.8 | 63.8 | 22.0 | 22.2 | 56.8 | 57.1 | NR | NR | 222 | 223 | NR | NR |
| Moya, 2016 | 61 | 61 | 69.0 | 68.0 | NR | NR | NR | NR | NR | NR | NR | NR | NR | NR |
| Moya, 2016 | 122 | 122 | 68.0 | 70.0 | 26.6 | 27.1 | NR | NR | NR | NR | NR | NR | NR | NR |
| Mudge, 2018 | 65 | 62 | 64.6 | 64.6 | 26.9 | 26.9 | NR | NR | NR | NR | 339 | 334 | 6 | 4 |
|  | 65 | 62 | 62.5 | 64.6 | 26.8 | 26.9 | NR | NR | NR | NR | 316 | 334 | 9 | 4 |
|  | 71 | 62 | 62.5 | 64.6 | 26.9 | 26.9 | NR | NR | NR | NR | 343 | 334 | 5 | 4 |
| Nakamura, 2005 | 12 | 14 | 64.0 | 64.0 | 19.0 | 19.0 | NR | NR | NR | NR | 598 | 476 | NR | NR |
| Okamoto, 2009 | 30 | 30 | 66.9 | 70.9 | NR | NR | NR | NR | NR | NR | 278 | 228 | 4 | 6 |
| Sakurai, 2007 | 16 | 14 | 63.0 | 63.0 | NR | NR | NR | NR | NR | NR | 455 | 411 | 1 | 1 |
| Scislo, 2018 | 44 | 54 | 62.6 | 62.9 | 24.7 | 25.1 | NR | NR | 9.1 | 12.5 | 216 | 225 | NR | NR |
| Wierdak, 2021 | 14 | 12 | 69.9 | 68.4 | 29.2 | 27.8 | NR | NR | NR | NR | NR | NR | NR | NR |
| Xu, 2006 | 30 | 30 | 60.1 | 57.7 | NR | NR | NR | NR | NR | NR | 192 | 180 | 7 | 9 |
| Yildiz, 2016 | 21 | 20 | 64.1 | 62.6 | 22.0 | 21.1 | NR | NR | NR | NR | 210 | 205 | 16 | 15 |

NR, not reported.
