# Supplementary material for: Effect of Enteral Immunonutrition in Patients Undergoing Surgery for Gastrointestinal Cancer: An Updated Systematic Review and Meta-Analysis
Source: Front Nutr. 2022 Jun 29;9:941975. doi: 10.3389/fnut.2022.941975 (PMC9277464; doi:10.3389/fnut.2022.941975)
Supplement: Supplementary Table 2 — Analysis of gastric cancer outcomes. [file Table_2.doc]

Supplementary Table 2. Analysis of [gastric](javascript:;) [cancer](javascript:;) outcomes.

| Enteral immunonutrition vs. Control | No. of studies | RR | 95%CI | *p* | Heterogeneity(I^2^) |
| --- | --- | --- | --- | --- | --- |
| Overall complications | 5 | 0.80 | 0.54, 1.18 | 0.26 | 38% |
| Infectious | | | | | |
| Infectious complications | 6 | 0.58 | 0.32, 1.05 | 0.07 | 51% |
| Surgical site infection | 6 | 0.85 | 0.56, 1.27 | 0.42 | 0% |
| Respiratory tract infection | 6 | 0.90 | 0.54, 1.49 | 0.67 | 0% |
| Urinary tract infection | 2 | 0.50 | 0.10, 2.67 | 0.42 | 0% |
| Abdominal abscess | 4 | 0.83 | 0.30, 2.32 | 0.72 | 11% |
| Infection of venous catheter | 2 | 0.99 | 0.15, 6.67 | 0.99 | 0% |
| Anastomotic leakage | 3 | 0.63 | 0.22, 1.86 | 0.41 | 0% |
| Sepsis | 2 | 0.34 | 0.04, 3.13 | 0.34 | 0% |
| Duration of SIRS | 2 | -0.89^*^ | -1.40, -0.39 | <0.001 | 53% |
| Non-infectious | | | | | |
| Non-infectious complications | 2 | 0.68 | 0.25, 1.80 | 0.43 | 0% |
| Intestinal obstruction | 2 | 2.20 | 0.33, 14.81 | 0.42 | 0% |
| Wound dehiscence | 2 | 0.74 | 0.05, 10.62 | 0.83 | 34% |
| Postoperative bleeding | 3 | 1.96 | 0.33, 11.56 | 0.46 | 0% |
| Length of hospital stay | 4 | -2.26^*^ | -4.69, 0.17 | 0.07 | 82% |
| Mortality | 3 | 0.39 | 0.09, 1.76 | 0.22 | 0% |
| Enteral nutrition related | | | | | |
| Adverse effects | 3 | 0.54 | 0.28, 1.05 | 0.07 | 0% |

^*^ indicates continuous data, using [mean difference](javascript:;).

RR, risk ratio; CI, confidence interval; SIRS, systemic inflammatory response syndrome.
