# Supplementary material for: Effect of Enteral Immunonutrition in Patients Undergoing Surgery for Gastrointestinal Cancer: An Updated Systematic Review and Meta-Analysis
Source: Front Nutr. 2022 Jun 29;9:941975. doi: 10.3389/fnut.2022.941975 (PMC9277464; doi:10.3389/fnut.2022.941975)
Supplement: Supplementary Table 3 — Analysis of colorectal cancer outcomes. [file Table_3.doc]

Supplementary Table 3. Analysis of colorectal cancer outcomes.

| Enteral immunonutrition vs. Control | No. of studies | RR | 95%CI | *p* | Heterogeneity(I^2^) |
| --- | --- | --- | --- | --- | --- |
| Overall complications | 4 | 0.83 | 0.63, 1.09 | 0.18 | 0% |
| Infectious | | | | | |
| Infectious complications | 5 | 0.50 | 0.36, 0.70 | <0.001 | 15% |
| Surgical site infection | 6 | 0.43 | 0.22, 0.81 | 0.009 | 32% |
| Respiratory tract infection | 5 | 0.62 | 0.31, 1.24 | 0.17 | 0% |
| Urinary tract infection | 5 | 0.69 | 0.35, 1.37 | 0.29 | 0% |
| Abdominal abscess | 2 | 0.44 | 0.09, 2.07 | 0.30 | 0% |
| Infection of venous catheter | 2 | 0.65 | 0.18, 2.34 | 0.51 | 20% |
| Anastomotic leakage | 5 | 0.61 | 0.36, 1.03 | 0.07 | 0% |
| Non-infectious | | | | | |
| Non-infectious complications | 3 | 1.15 | 0.71, 1.87 | 0.58 | 0% |
| Intestinal obstruction | 6 | 0.80 | 0.48, 1.31 | 0.37 | 0% |
| Length of hospital stay | 3 | -1.74^*^ | -3.23, -0.25 | 0.02 | 83% |

^*^ indicates continuous data, using [mean difference](javascript:;).

RR, risk ratio; CI, confidence interval.
