# Supplementary material for: Effect of Enteral Immunonutrition in Patients Undergoing Surgery for Gastrointestinal Cancer: An Updated Systematic Review and Meta-Analysis
Source: Front Nutr. 2022 Jun 29;9:941975. doi: 10.3389/fnut.2022.941975 (PMC9277464; doi:10.3389/fnut.2022.941975)
Supplement: Supplementary Table 4 — Analysis of esophageal cancer outcomes. [file Table_4.doc]

Supplementary Table 4. Analysis of oesophageal cancer outcomes.

| Enteral immunonutrition vs. Control | No. of studies | RR | 95%CI | *p* | Heterogeneity(I^2^) |
| --- | --- | --- | --- | --- | --- |
| Infectious | | | | | |
| Infectious complications | 4 | 0.95 | 0.70, 1.29 | 0.73 | 36% |
| Surgical site infection | 5 | 1.05 | 0.55, 2.00 | 0.89 | 23% |
| Respiratory tract infection | 5 | 1.12 | 0.79, 1.60 | 0.52 | 7% |
| Anastomotic leakage | 4 | 0.80 | 0.48, 1.33 | 0.39 | 0% |
| Length of hospital stay | 3 | -1.45^*^ | -3.86, 0.95 | 0.24 | 0% |

^*^ indicates continuous data, using [mean difference](javascript:;).

RR, risk ratio; CI, confidence interval.
