# Supplementary material for: Effect of Enteral Immunonutrition in Patients Undergoing Surgery for Gastrointestinal Cancer: An Updated Systematic Review and Meta-Analysis
Source: Front Nutr. 2022 Jun 29;9:941975. doi: 10.3389/fnut.2022.941975 (PMC9277464; doi:10.3389/fnut.2022.941975)
Supplement: Supplementary Table 5 — Analysis of periampullary cancer (including pancreatic cancer) outcomes. [file Table_5.doc]

Supplementary Table 5. Analysis of [periampullary](javascript:;) cancer (including pancreatic cancer) outcomes.

| Enteral immunonutrition vs. Control | No. of studies | RR | 95%CI | *p* | Heterogeneity(I^2^) |
| --- | --- | --- | --- | --- | --- |
| Infectious | | | | | |
| Infectious complications | 2 | 0.57 | 0.33, 1.01 | 0.05 | 0% |
| Surgical site infection | 2 | 0.36 | 0.16, 0.84 | 0.02 | 0% |
| Respiratory tract infection | 2 | 0.57 | 0.02, 13.24 | 0.72 | 54% |
| Abdominal abscess | 2 | 0.55 | 0.25, 1.21 | 0.14 | 0% |
| Sepsis | 2 | 0.57 | 0.07, 4.41 | 0.59 | 0% |
| Non-infectious | | | | | |
| Non-infectious complications | 2 | 0.90 | 0.62, 1.32 | 0.60 | 0% |
| Postoperative bleeding | 2 | 0.46 | 0.08, 2.50 | 0.37 | 0% |

RR, risk ratio; CI, confidence interval.
