# Supplementary material for: Effect of Enteral Immunonutrition in Patients Undergoing Surgery for Gastrointestinal Cancer: An Updated Systematic Review and Meta-Analysis
Source: Front Nutr. 2022 Jun 29;9:941975. doi: 10.3389/fnut.2022.941975 (PMC9277464; doi:10.3389/fnut.2022.941975)
Supplement: Supplementary Table 6 — Analysis of preoperative nutrition outcomes. [file Table_6.doc]

Supplementary Table 6. Analysis of preoperative nutrition outcomes.

| Enteral immunonutrition vs. Control | No. of studies | RR | 95%CI | *p* | Heterogeneity(I^2^) |
| --- | --- | --- | --- | --- | --- |
| Overall complications | 9 | 0.87 | 0.72, 1.05 | 0.15 | 3% |
| Infectious | | | | | |
| Infectious complications | 12 | 0.62 | 0.48, 0.81 | <0.001 | 36% |
| Surgical site infection | 15 | 0.79 | 0.56, 1.13 | 0.19 | 20% |
| Respiratory tract infection | 14 | 0.72 | 0.48, 1.07 | 0.10 | 0% |
| Urinary tract infection | 10 | 0.72 | 0.42, 1.25 | 0.25 | 0% |
| Respiratory failure | 6 | 0.99 | 0.48, 2.04 | 0.98 | 0% |
| Abdominal abscess | 10 | 0.66 | 0.41, 1.06 | 0.08 | 0% |
| Infection of venous catheter | 2 | 0.99 | 0.15, 6.67 | 0.99 | 0% |
| Pancreatic fistula | 5 | 0.95 | 0.53, 1.69 | 0.86 | 0% |
| Anastomotic leakage | 10 | 0.65 | 0.42, 0.99 | 0.04 | 0% |
| Bacteremia | 3 | 0.30 | 0.09, 0.98 | 0.05 | 0% |
| Sepsis | 6 | 0.40 | 0.15, 1.11 | 0.08 | 0% |
| SIRS | 3 | 1.22 | 0.86, 1.73 | 0.27 | 0% |
| Duration of SIRS | 3 | -0.27^*^ | -0.33, -0.21 | <0.001 | 49% |
| Duration of antibiotic therapy | 4 | -2.32^*^ | -3.20, -1.44 | <0.001 | 72% |
| Non-infectious | | | | | |
| Non-infectious complications | 11 | 0.95 | 0.79, 1.13 | 0.54 | 0% |
| Vein thrombosis | 3 | 0.66 | 0.11, 4.13 | 0.66 | 0% |
| Arrythmia | 2 | 0.60 | 0.07, 5.46 | 0.65 | 49% |
| Cardiac dysfunction | 3 | 0.75 | 0.19, 3.06 | 0.69 | 0% |
| Renal dysfunction | 5 | 1.40 | 0.50, 3.93 | 0.52 | 0% |
| Delayed gastric emptying | 4 | 1.46 | 0.69, 3.12 | 0.32 | 0% |
| Intestinal obstruction | 10 | 1.10 | 0.56, 2.15 | 0.78 | 0% |
| Wound dehiscence | 2 | 1.19 | 0.34, 4.17 | 0.79 | 0% |
| Postoperative bleeding | 9 | 0.66 | 0.28, 1.55 | 0.34 | 0% |
| Pleural effusion | 4 | 0.83 | 0.35, 1.95 | 0.67 | 0% |
| Length of hospital stay | 12 | -2.10^*^ | -3.62, -0.58 | 0.007 | 85% |
| Mortality | 7 | 0.87 | 0.31, 2.46 | 0.79 | 0% |
| Enteral nutrition related | | | | | |
| Vomiting | 2 | 1.52 | 0.30, 7.77 | 0.61 | 30% |
| Diarrhoea | 4 | 0.65 | 0.35, 1.21 | 0.17 | 0% |

^*^ indicates continuous data, using [mean difference](javascript:;).

RR, risk ratio; CI, confidence interval; SIRS, systemic inflammatory response syndrome.
