# Supplementary material for: Effect of Enteral Immunonutrition in Patients Undergoing Surgery for Gastrointestinal Cancer: An Updated Systematic Review and Meta-Analysis
Source: Front Nutr. 2022 Jun 29;9:941975. doi: 10.3389/fnut.2022.941975 (PMC9277464; doi:10.3389/fnut.2022.941975)
Supplement: Supplementary Table 7 — Analysis of postoperative nutrition outcomes. [file Table_7.doc]

Supplementary Table 7. Analysis of postoperative nutrition outcomes.

| Enteral immunonutrition vs. Control | No. of studies | RR | 95%CI | *p* | Heterogeneity(I^2^) |
| --- | --- | --- | --- | --- | --- |
| Overall complications | 7 | 0.80 | 0.66, 0.96 | 0.02 | 0% |
| Infectious | | | | | |
| Infectious complications | 8 | 0.84 | 0.67, 1.05 | 0.13 | 20% |
| Surgical site infection | 8 | 0.61 | 0.40, 0.93 | 0.02 | 0% |
| Respiratory tract infection | 10 | 0.93 | 0.75, 1.15 | 0.50 | 0% |
| Urinary tract infection | 6 | 0.79 | 0.44, 1.43 | 0.43 | 0% |
| Respiratory failure | 2 | 0.75 | 0.26, 2.12 | 0.59 | 0% |
| Abdominal abscess | 6 | 0.65 | 0.31, 1.37 | 0.26 | 0% |
| Infection of venous catheter | 2 | 1.40 | 0.10, 20.53 | 0.80 | 34% |
| Pancreatic fistula | 3 | 0.57 | 0.25, 1.32 | 0.19 | 0% |
| Duodenal fistula | 4 | 1.24 | 0.38, 3.97 | 0.72 | 0% |
| Anastomotic leakage | 4 | 0.81 | 0.45, 1.48 | 0.50 | 0% |
| Bacteremia | 3 | 0.43 | 0.19, 0.97 | 0.04 | 0% |
| Sepsis | 5 | 0.75 | 0.34, 1.66 | 0.47 | 0% |
| Non-infectious | | | | | |
| Non-infectious complications | 2 | 0.91 | 0.66, 1.25 | 0.56 | 32% |
| Vein thrombosis | 2 | 0.98 | 0.14, 6.59 | 0.98 | 0% |
| Pulmonary thrombosis | 2 | 0.35 | 0.05, 2.29 | 0.27 | 0% |
| Myocardial infarction | 2 | 3.01 | 0.32, 28.59 | 0.34 | 0% |
| Cardiac dysfunction | 3 | 0.52 | 0.13, 2.08 | 0.35 | 0% |
| Wound dehiscence | 5 | 0.38 | 0.13, 1.08 | 0.07 | 0% |
| Postoperative bleeding | 2 | 1.00 | 0.30, 3.38 | 1.00 | 0% |
| Length of hospital stay | 5 | -1.70^*^ | -3.72, 0.32 | 0.10 | 89% |
| Mortality | 9 | 0.54 | 0.28, 1.07 | 0.08 | 0% |
| Enteral nutrition related | | | | | |
| Adverse effects | 3 | 0.74 | 0.45, 1.21 | 0.24 | 18% |
| Diarrhoea | 3 | 0.74 | 0.42, 1.29 | 0.29 | 0% |

^*^ indicates continuous data, using [mean difference](javascript:;).

RR, risk ratio; CI, confidence interval.
