# Supplementary material for: Effect of Enteral Immunonutrition in Patients Undergoing Surgery for Gastrointestinal Cancer: An Updated Systematic Review and Meta-Analysis
Source: Front Nutr. 2022 Jun 29;9:941975. doi: 10.3389/fnut.2022.941975 (PMC9277464; doi:10.3389/fnut.2022.941975)
Supplement: Supplementary Table 8 — Analysis of standard diet as control groups. [file Table_8.doc]

Supplementary Table 8. Analysis of standard diet as control groups.

| Enteral immunonutrition vs. Control | No. of studies | RR | 95%CI | *p* | Heterogeneity(I^2^) |
| --- | --- | --- | --- | --- | --- |
| Overall complications | 13 | 0.78 | 0.66, 0.92 | 0.003 | 4% |
| Infectious | | | | | |
| Infectious complications | 18 | 0.73 | 0.59, 0.90 | 0.003 | 45% |
| Surgical site infection | 19 | 0.68 | 0.49, 0.94 | 0.02 | 11% |
| Respiratory tract infection | 21 | 0.91 | 0.76, 1.09 | 0.31 | 0% |
| Urinary tract infection | 12 | 0.71 | 0.44, 1.13 | 0.15 | 0% |
| Respiratory failure | 5 | 0.74 | 0.35, 1.56 | 0.42 | 0% |
| Abdominal abscess | 12 | 0.53 | 0.29, 1.00 | 0.05 | 0% |
| Infection of venous catheter | 5 | 0.53 | 0.22, 1.31 | 0.17 | 0% |
| Pancreatic fistula | 5 | 0.58 | 0.27, 1.24 | 0.16 | 0% |
| Duodenal fistula | 4 | 1.24 | 0.38, 3.97 | 0.72 | 0% |
| Anastomotic leakage | 10 | 0.71 | 0.49, 1.02 | 0.07 | 0% |
| Bacteremia | 4 | 0.42 | 0.20, 0.90 | 0.02 | 0% |
| Sepsis | 9 | 0.72 | 0.42, 1.23 | 0.23 | 0% |
| Duration of SIRS | 2 | -0.89^*^ | -1.40, -0.39 | <0.001 | 53% |
| Duration of antibiotic therapy | 2 | -1.43^*^ | -3.57, 0.71 | 0.19 | 84% |
| Non-infectious | | | | | |
| Non-infectious complications | 9 | 0.94 | 0.81, 1.10 | 0.45 | 0% |
| Vein thrombosis | 3 | 0.73 | 0.14, 3.77 | 0.71 | 0% |
| Pulmonary thrombosis | 3 | 0.34 | 0.07, 1.73 | 0.20 | 0% |
| Arrythmia | 2 | 0.89 | 0.36, 2.18 | 0.80 | 0% |
| Myocardial infarction | 3 | 2.97 | 0.47, 18.65 | 0.25 | 0% |
| Cardiac dysfunction | 5 | 0.60 | 0.21, 1.72 | 0.34 | 0% |
| Renal dysfunction | 4 | 1.90 | 0.65, 5.57 | 0.24 | 0% |
| Delayed gastric emptying | 4 | 0.67 | 0.34, 1.35 | 0.27 | 0% |
| Intestinal obstruction | 5 | 0.74 | 0.39, 1.40 | 0.35 | 0% |
| Wound dehiscence | 9 | 0.58 | 0.28, 1.22 | 0.15 | 0% |
| Postoperative bleeding | 6 | 0.99 | 0.38, 2.60 | 0.99 | 0% |
| Pleural effusion | 2 | 1.19 | 0.36, 3.94 | 0.77 | 0% |
| Length of hospital stay | 14 | -1.28^*^ | -2.42, -0.13 | 0.03 | 68% |
| Mortality | 11 | 0.64 | 0.36, 1.16 | 0.14 | 0% |
| Enteral nutrition related | | | | | |
| Adverse effects | 7 | 0.91 | 0.73, 1.13 | 0.38 | 0% |
| Bloating | 4 | 0.85 | 0.48, 1.49 | 0.57 | 0% |
| Vomiting | 4 | 1.36 | 0.69, 2.68 | 0.37 | 0% |
| Diarrhoea | 7 | 0.72 | 0.46, 1.14 | 0.16 | 0% |

^*^ indicates continuous data, using [mean difference](javascript:;).

RR, risk ratio; CI, confidence interval; SIRS, systemic inflammatory response syndrome.
