# Supplementary material for: Effect of Enteral Immunonutrition in Patients Undergoing Surgery for Gastrointestinal Cancer: An Updated Systematic Review and Meta-Analysis
Source: Front Nutr. 2022 Jun 29;9:941975. doi: 10.3389/fnut.2022.941975 (PMC9277464; doi:10.3389/fnut.2022.941975)
Supplement: Supplementary Table 9 — Analysis of no supplement as control groups. [file Table_9.doc]

Supplementary Table 9. Analysis of no supplement as control groups.

| Enteral immunonutrition vs. Control | No. of studies | RR | 95%CI | *p* | Heterogeneity(I^2^) |
| --- | --- | --- | --- | --- | --- |
| Overall complications | 8 | 0.80 | 0.67, 0.94 | 0.009 | 8% |
| Infectious | | | | | |
| Infectious complications | 9 | 0.55 | 0.43, 0.72 | <0.001 | 31% |
| Surgical site infection | 12 | 0.63 | 0.45, 0.89 | 0.008 | 18% |
| Respiratory tract infection | 11 | 0.74 | 0.47, 1.14 | 0.17 | 0% |
| Urinary tract infection | 6 | 0.71 | 0.40, 1.26 | 0.25 | 0% |
| Respiratory failure | 4 | 1.14 | 0.63, 2.06 | 0.66 | 0% |
| Abdominal abscess | 7 | 0.63 | 0.40, 0.99 | 0.04 | 0% |
| Infection of venous catheter | 2 | 1.92 | 0.36, 10.36 | 0.45 | 0% |
| Pancreatic fistula | 4 | 1.07 | 0.64, 1.78 | 0.80 | 0% |
| Anastomotic leakage | 9 | 0.59 | 0.39, 0.88 | 0.01 | 0% |
| Bacteremia | 3 | 0.26 | 0.10, 0.70 | 0.008 | 0% |
| Sepsis | 3 | 0.44 | 0.11, 1.75 | 0.24 | 0% |
| SIRS | 3 | 1.20 | 0.84, 1.69 | 0.31 | 0% |
| Duration of SIRS | 2 | -0.27^*^ | -0.33, -0.21 | <0.001 | 60% |
| Duration of antibiotic therapy | 3 | -2.79^*^ | -3.49, -2.08 | <0.001 | 59% |
| Non-infectious | | | | | |
| Non-infectious complications | 8 | 0.85 | 0.70, 1.03 | 0.09 | 0% |
| Vein thrombosis | 2 | 0.66 | 0.11, 4.13 | 0.66 | 0% |
| Arrythmia | 2 | 0.65 | 0.16, 2.62 | 0.55 | 4% |
| Cardiac dysfunction | 2 | 1.43 | 0.18, 11.16 | 0.73 | 0% |
| Renal dysfunction | 4 | 0.70 | 0.19, 2.59 | 0.59 | 0% |
| Delayed gastric emptying | 3 | 1.25 | 0.67, 2.34 | 0.48 | 0% |
| Intestinal obstruction | 9 | 1.05 | 0.58, 1.90 | 0.88 | 0% |
| Wound dehiscence | 2 | 0.86 | 0.26, 2.84 | 0.80 | 0% |
| Postoperative bleeding | 7 | 0.53 | 0.24, 1.16 | 0.11 | 0% |
| Pleural effusion | 3 | 0.56 | 0.22, 1.42 | 0.22 | 0% |
| Length of hospital stay | 9 | -2.71^*^ | -4.20, -1.22 | <0.001 | 89% |
| Mortality | 5 | 0.75 | 0.28, 2.01 | 0.57 | 0% |
| Enteral nutrition related | | | | | |
| Diarrhoea | 3 | 0.97 | 0.55, 1.73 | 0.93 | 0% |

^*^ indicates continuous data, using [mean difference](javascript:;).

RR, risk ratio; CI, confidence interval; SIRS, systemic inflammatory response syndrome.
