# Supplementary material for: Effect of Enteral Immunonutrition in Patients Undergoing Surgery for Gastrointestinal Cancer: An Updated Systematic Review and Meta-Analysis
Source: Front Nutr. 2022 Jun 29;9:941975. doi: 10.3389/fnut.2022.941975 (PMC9277464; doi:10.3389/fnut.2022.941975)
Supplement: Supplementary Table 10 — Analysis of malnourished patients outcomes. [file Table_10.doc]

Supplementary Table 10. Analysis of malnourished patients outcomes.

| Enteral immunonutrition vs. Control | No. of studies | RR | 95%CI | *p* | Heterogeneity(I^2^) |
| --- | --- | --- | --- | --- | --- |
| Overall complications | 3 | 0.67 | 0.54, 0.84 | <0.001 | 0% |
| Infectious | | | | | |
| Infectious complications | 3 | 0.71 | 0.54, 0.92 | 0.01 | 0% |
| Surgical site infection | 3 | 0.69 | 0.38, 1.25 | 0.22 | 21% |
| Respiratory tract infection | 4 | 0.75 | 0.55, 1.02 | 0.07 | 0% |
| Urinary tract infection | 3 | 0.71 | 0.37, 1.37 | 0.31 | 0% |
| Respiratory failure | 2 | 0.64 | 0.25, 1.63 | 0.35 | 0% |
| Abdominal abscess | 3 | 0.48 | 0.20, 1.19 | 0.11 | 0% |
| Pancreatic fistula | 2 | 0.59 | 0.22, 1.53 | 0.28 | 0% |
| Duodenal fistula | 2 | 1.07 | 0.16, 7.20 | 0.95 | 0% |
| Bacteremia | 3 | 0.28 | 0.10, 0.78 | 0.02 | 0% |
| Sepsis | 3 | 0.85 | 0.24, 3.02 | 0.80 | 5% |
| Non-infectious | | | | | |
| Non-infectious complications | 2 | 0.91 | 0.55, 1.48 | 0.69 | 0% |
| Vein thrombosis | 3 | 0.62 | 0.15, 2.67 | 0.52 | 0% |
| Cardiac dysfunction | 2 | 0.57 | 0.12, 2.70 | 0.48 | 0% |
| Renal dysfunction | 2 | 4.74 | 0.54, 41.27 | 0.16 | 0% |
| Delayed gastric emptying | 2 | 0.71 | 0.34, 1.47 | 0.35 | 0% |
| Intestinal obstruction | 2 | 1.36 | 0.26, 7.13 | 0.72 | 0% |
| Wound dehiscence | 2 | 0.27 | 0.07, 1.06 | 0.06 | 0% |
| Postoperative bleeding | 2 | 0.48 | 0.11, 2.03 | 0.32 | 0% |
| Length of hospital stay | 3 | -2.62^*^ | -4.04, -1.19 | <0.001 | 31% |
| Mortality | 3 | 0.26 | 0.09, 0.78 | 0.02 | 0% |

^*^ indicates continuous data, using [mean difference](javascript:;).

RR, risk ratio; CI, confidence interval.
