# Supplementary material for: Effect of Enteral Immunonutrition in Patients Undergoing Surgery for Gastrointestinal Cancer: An Updated Systematic Review and Meta-Analysis
Source: Front Nutr. 2022 Jun 29;9:941975. doi: 10.3389/fnut.2022.941975 (PMC9277464; doi:10.3389/fnut.2022.941975)
Supplement: Supplementary Table 11 — Analysis of well-nourished patients outcomes. [file Table_11.doc]

Supplementary Table 11. Analysis of well-nourished patients outcomes.

| Enteral immunonutrition vs. Control | No. of studies | RR | 95%CI | *p* | Heterogeneity(I^2^) |
| --- | --- | --- | --- | --- | --- |
| Overall complications | 2 | 0.75 | 0.60, 0.93 | 0.01 | 0% |
| Infectious | | | | | |
| Infectious complications | 3 | 0.51 | 0.37, 0.71 | <0.001 | 0% |
| Surgical site infection | 4 | 0.56 | 0.33, 0.95 | 0.03 | 0% |
| Respiratory tract infection | 4 | 0.54 | 0.28, 1.02 | 0.06 | 0% |
| Urinary tract infection | 3 | 0.72 | 0.31, 1.67 | 0.44 | 0% |
| Respiratory failure | 2 | 1.17 | 0.57, 2.40 | 0.67 | 0% |
| Abdominal abscess | 2 | 0.41 | 0.19, 0.87 | 0.02 | 0% |
| Pancreatic fistula | 2 | 1.27 | 0.59, 2.76 | 0.54 | 0% |
| Anastomotic leakage | 3 | 0.44 | 0.21, 0.91 | 0.03 | 0% |
| Sepsis | 2 | 0.61 | 0.12, 3.05 | 0.55 | 0% |
| Non-infectious | | | | | |
| Non-infectious complications | 2 | 0.84 | 0.64, 1.09 | 0.19 | 0% |
| Pulmonary thrombosis | 2 | 0.98 | 0.10, 9.34 | 0.99 | 0% |
| Renal dysfunction | 2 | 0.91 | 0.26, 3.17 | 0.89 | 0% |
| Delayed gastric emptying | 2 | 1.27 | 0.58, 2.81 | 0.55 | 0% |
| Intestinal obstruction | 2 | 0.75 | 0.14, 3.95 | 0.74 | 0% |
| Postoperative bleeding | 2 | 0.38 | 0.11, 1.28 | 0.12 | 0% |
| Pleural effusion | 2 | 0.74 | 0.26, 2.12 | 0.57 | 16% |
| Length of hospital stay | 3 | -1.36^*^ | -2.61, -0.11 | 0.03 | 38% |
| Mortality | 2 | 1.64 | 0.39, 6.90 | 0.50 | 0% |
| Enteral nutrition related | | | | | |
| Vomiting | 2 | 1.44 | 0.49, 4.25 | 0.51 | 0% |
| Diarrhoea | 2 | 1.03 | 0.31, 3.44 | 0.96 | 38% |

^*^ indicates continuous data, using [mean difference](javascript:;).

RR, risk ratio; CI, confidence interval.
